# Pathological complete response as a surrogate to improved survival in human epidermal growth factor receptor-2-positive breast cancer: systematic review and meta-analysis

**DOI:** 10.1093/bjsopen/zrac028

**Published:** 2022-05-04

**Authors:** Matthew G. Davey, Ferdia Browne, Nicola Miller, Aoife J. Lowery, Michael J. Kerin

**Affiliations:** 1 Department of Surgery, Galway University Hospitals, Galway, Ireland; 2 The Lambe Institute for Translational Research, National University of Ireland, Galway, Ireland

## Abstract

**Background:**

Achieving a pathological complete response (pCR) is believed to correlate with oncological outcomes in human epidermal growth factor receptor-2-positive (HER2^+^) breast cancer. However, informed estimation of this survival advantage is often difficult to quantify. The aim of this study was to evaluate the role of pCR as a biomarker of survival in patients treated with neoadjuvant therapies for HER2^+^ breast cancer.

**Methods:**

A systematic review was performed in accordance with the PRISMA checklist. Data specific to pCR and survival with respect to event-free survival (EFS), recurrence-free survival (RFS) and overall survival (OS) were expressed as hazard ratio (HR) and 95 per cent confidence intervals (c.i.). pCR and survival at yearly intervals after resection were expressed as dichotomous variables using the Mantel–Haenszel method.

**Results:**

Overall, 78 clinical studies with 25 150 patients were included in this study. pCR predicted better EFS (HR 0.67, 95 per cent c.i. 0.60 to 0.74; 41 studies), RFS (HR 0.69, 95 per cent c.i. 0.57 to 0.83; 18 studies) and OS (HR 0.63, 95 per cent c.i. 0.56 to 0.70; 29 studies) for patients with HER2^+^ breast cancer. At 5 years, pCR predicted better EFS (HR 0.37, 95 per cent c.i. 0.30 to 0.48; 19 studies), RFS (HR 0.28, 95 per cent c.i. 0.21 to 0.39; 8 studies) and OS (HR 0.26, 95 per cent c.i. 0.20 to 0.33; 10 studies).

**Conclusion:**

This study confirms pCR as an informative surrogate biomarker for enhanced survival and suggests that it may be used as an appropriate endpoint for clinical research.

## Teaser

This meta-analysis, which includes data from 78 studies, successfully highlights and quantifies the positive prognostic value for those achieving a pathological complete response *versus* those with residual disease following the prescription of neoadjuvant therapies for human epidermal growth factor receptor-2-positive breast cancers.

## Introduction

In recent years, neoadjuvant chemotherapy (NAC) has become an established facet of multidisciplinary management of patients with breast cancer^1^. Survival outcomes in patients treated with NAC are similar to those treated with adjuvant chemotherapy (AC)^2^. Despite similar outcomes, NAC is advantageous as it has the ability to make previously inoperable tumours resectable, improves patient eligibility for breast conservation surgery, and provides *in vivo* data indicating the sensitivity of tumours to conventional therapeutic strategies^3^. When quantifying responses to NAC, the complete eradication of cancer cells following treatment is referred to as pathological complete response (pCR)^4^. Achieving pCR is believed to correlate with enhanced oncological outcomes, although the estimation of this survival advantage is difficult to quantify. Moreover, the benefit of pCR is perceived to vary among each of the four molecular breast cancer subtypes, further casting uncertainty in relation to the value of pCR in gauging prognosis^5^.

Approximately 20–25 per cent of breast cancers possess amplification of the human epidermal growth factor receptor-2 (*HER2*/neu) gene, which is critical in tumour proliferation and disease progression^6^. These cancers tend to harbour aggressive clinicopathological features and were traditionally associated with poor clinical outcomes^7^. According to the recent American Society of Clinical Oncology/College of American Pathologists (ASCO/CAP) guidelines (2021), most HER2-positive (HER2^+^) breast cancers should be considered for NAC, with exceptions limited to only those with T1a N0 or T1b N0 disease (unless in the clinical trial setting)^8^. With the increased propensity to prescribe NAC to this group, the aim of the present study was to perform a systematic review and meta-analysis evaluating the role of pCR as a biomarker of survival in patients treated with NAC for HER2^+^ breast cancer.

## Methods

A systematic review was performed in accordance to the PRISMA checklist^9^ and meta-analysis and systematic review s of observational studies (MOOSE) guidelines^10^. Local institutional ethical approval was not required and this study was registered with the International Prospective Register of Systematic Reviews (PROSPERO: CRD42021284195).

### Search strategy

An electronic search was performed of the *PubMed Medline*, *EMBASE* and *Scopus* databases on 14 February 2021 for relevant studies that would be suitable for inclusion in this study. The search was performed of all fields under the following headings: ((((‘breast cancer’) AND (‘HER2’)) AND (‘pathological complete response’)) AND (‘neoadjuvant therapy’)) AND (‘survival’). Included studies were limited to those published in the English language, on account of the challenges outlined in depth by Neimann Rasmussen *et al*. in their article (lack of resources (funding and time) and lack of language resources, such as lack of translators)^11^. Included studies were not restricted based on year of publication. All titles were initially screened, and studies deemed appropriate had their abstracts and full texts reviewed.

### Inclusion and exclusion criteria

Studies meeting the following inclusion criteria were included: studies with patients with histologically confirmed HER2^+^ primary breast cancer (either HER2^+^ luminal B or HER2-enriched breast cancer molecular subtypes)^12^; studies investigating the correlation between pCR and survival outcomes (event-free survival (EFS), recurrence-free survival (RFS) or overall survival (OS)). Clinicopathological parameters and treatment characteristics were also recorded and correlated with pCR. Studies meeting any of the following exclusion criteria were excluded from this study: studies failing to outline pCR as an indicator of survival in HER2^+^ breast cancer; studies outlining pCR as a biomarker of survival in other breast cancer molecular subtypes (triple negative or luminal cancers) or in which no distinction has been made for molecular subtyping; review articles; studies including fewer than five patients in their series or case reports; or editorial articles.

### Data extraction and quality assessment

The literature search was performed by two independent reviewers (M.G.D. and F.B.) by use of a predesigned search strategy. Duplicate studies were manually removed. Each reviewer then reviewed the titles, abstracts and/or full texts of the retrieved manuscripts to ensure that all inclusion criteria was met before extracting the following data:

- first author name;
- year of publication;
- study design;
- level of evidence;
- study title;
- number of patients;
- number of patients who successfully achieved a pCR and those with residual disease (RD);
- survival outcomes for EFS, RFS, or OS at yearly intervals after treatment; and
- neoadjuvant treatment characteristics.

Data specific to patient outcomes and survival (expressed as hazard ratio (HR), 95 per cent confidence intervals (c.i.) and *P* values) were directly extracted from tables and study text. HR and associated standard errors were calculated from Kaplan–Meier curves where relevant. Risk of bias and methodology quality assessment was performed in concordance with the Newcastle–Ottawa scale^13^. In case of discrepancies in opinion between the reviewers, a third reviewer was asked to arbitrate.

### Definitions

- NAC was defined as any systemic treatment given before surgery^14^, which included both cytotoxic chemotherapies and targeted therapies (anti-HER2 therapies, such as trastuzumab).
- pCR was defined as ‘no evidence of invasive and/or *in situ* disease in the breast and/or axillary lymph nodes’. Accepted definitions included residual *in situ* disease after NAC^4^.
- EFS was defined as ‘freedom from disease recurrence or progression, a second primary breast cancer or death’. The term EFS was preferred over disease-free survival as it included patients were not considered ‘disease-free’ at the time of neoadjuvant treatment (this is the case in those treated with adjuvant therapies).
- RFS was defined as ‘freedom from disease recurrence of the index cancer or death’. This included studies describing patients with relapse-free or recurrence-free intervals or survival.
- OS was defined as ‘death due to any cause, including breast cancer-related mortality’.

### Statistical analysis

Clinicopathological characteristics for those achieving pCR and those with RD were presented as proportions with descriptive statistics at yearly intervals (such as 2 years after resection and 3 years after resection). RFS, EFS, and OS for those achieving pCR were expressed as hazard ratios and were considered the primary analytical endpoints. Hazard ratios and each corresponding 95 per cent confidence intervals were retrieved from multivariable analyses when available. Alternatively, Kaplan­–Meier analyses were used to calculate the hazard ratios and respective standard errors. The impact of achieving pCR with respect to RFS, EFS, and OS at yearly intervals after resection were expressed as dichotomous variables using the Mantel–Haenszel method. Either fixed or random-effects models were applied on the basis of whether significant heterogeneity (*I*^2^ > 50 per cent) existed between studies included in analysis. Symmetry of funnel plots were used to assess publication bias. Statistical heterogeneity was determined using *I*^2^ statistics. Statistical significance was determined to be *P* < 0.050. Statistical analysis was performed with Review Manager (RevMan), version 5.4 (Nordic Cochrane Centre, Copenhagen, Denmark).

## Results

### Literature search

**Fig. 1:**
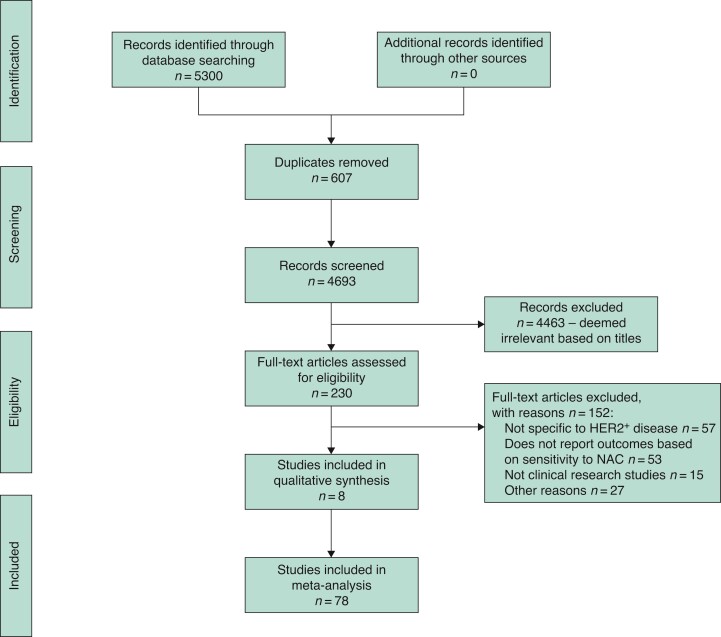
PRISMA flow diagram detailing the systematic search process

The initial electronic search resulted in a total of 5300 studies. Following removal of 607 duplicate studies, the remaining 4693 titles were screened for relevance, of which 230 had their abstracts and full texts assessed for eligibility. Overall, 78 clinical studies were included in this systematic review^15–92^, as depicted in *Fig. 1*. Individual studies included in this analysis are outlined in *Table S1*.

### Study characteristics

Overall, 25 150 patients were included in this study. Of these, 10 280 successfully achieved pCR after NAC (40.9 per cent) and 14 864 had RD after NAC (59.1 per cent). Molecular subtype was available for 17 973 patients; 9355 were HER2^+^ luminal B (52.1 per cent) and 8618 were HER2-enriched breast cancer molecular subtypes (47.9 per cent). pCR and its correlations with clinicopathological data are outlined in *Table S2*.

### Pathological complete response and event-free survival

**Fig. 2:**
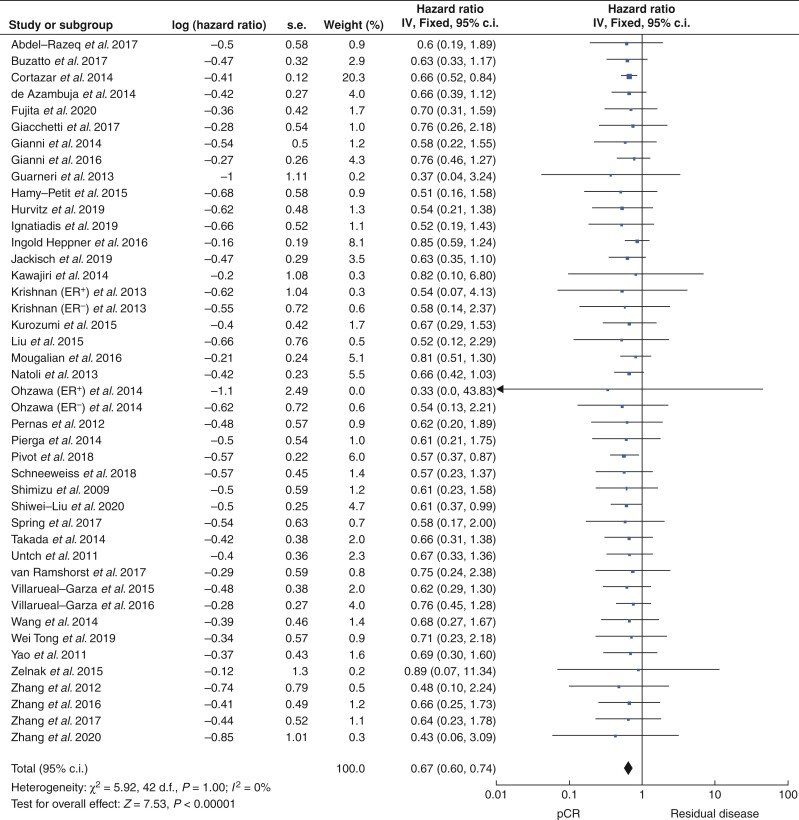
Forest plot comparing the hazards ratios relevant to event-free survival for patients who successfully achieved pathological complete response following neoadjuvant therapies *versus* those with residual disease

**Fig. 3:**
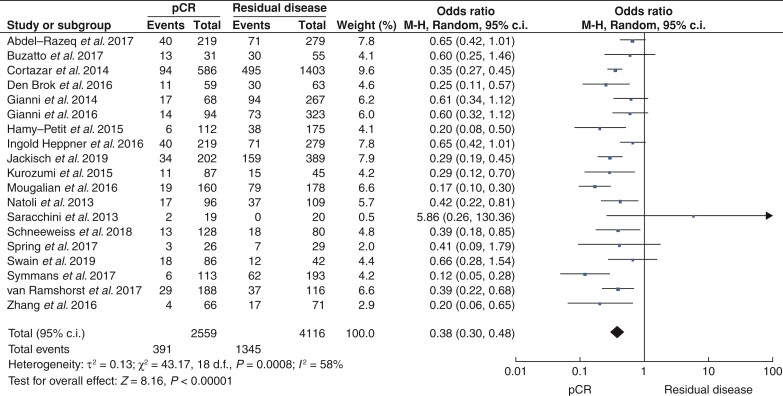
Forest plot comparing 5-year event-free survival for patients who successfully achieved pathological complete response following neoadjuvant therapies *versus* those with residual disease

Overall, 51 studies that included 12 535 patients reported outcomes in relation to pCR as an indicator of EFS. Of these, 41.1 per cent successfully achieved pCR (5153 patients) *versus* 58.9 per cent with RD (7382 patients). pCR was associated with better EFS annually after treatment (all *P* < 0.001, Fisher’s exact test) (*Table 1*). pCR predicted better EFS for patients with HER2^+^ breast cancer (HR 0.67, 95 per cent c.i. 0.60 to 0.74, *P* < 0.001, *I^2^* = 0 per cent; 41 studies) (*Fig. 2* and *Fig. S1*). pCR also predicted better EFS after 2 years (HR 0.28, 95 per cent c.i. 0.14 to 0.58, *P* < 0.001, *I^2^* = 34 per cent; 4 studies) (*Fig. S2*), after 3 years (HR 0.23, 95 per cent c.i. 0.14 to 0.37, *P* < 0.001, *I^2^* = 58 per cent; 10 studies) (*Fig. S3*), after 4 years (HR 0.28, 95 per cent c.i. 0.13 to 0.62, *P* < 0.001, *I^2^* = 0 per cent; 3 studies) (*Fig. S4*), after 5 years (HR 0.37, 95 per cent c.i. 0.30 to 0.48, *P* < 0.001, *I^2^* = 58 per cent; 19 studies) (*Fig. 3* and *Fig. S5*), and after 10 years (HR 0.10, 95 per cent c.i. 0.07 to 0.16, *P* < 0.001, *I^2^* = 7 per cent; 2 studies) (*Fig. S6*).

**Table 1:** Pathological complete response and residual disease and their correlation with clinical outcomes at annual intervals

Parameter	pCR	RD	*P*
**EFS**			
2-year event	12	50	<0.001*^†^
2-year EFS	173	210	
3-year event	86	390	<0.001*^†^
3-year EFS	1029	1094	
4-year event	13	37	<0.001*^†^
4-year EFS	141	131	
5-year event	346	1309	<0.001*^†^
5-year EFS	2026	2515	
10-year event	29	188	<0.001*^†^
10-year EFS	244	176	
**RFS**			
2-year event	4	3	0.430^†^
2-year RFS	21	33	
3-year event	11	50	<0.001*^†^
3-year RFS	177	160	
4-year event	8	34	<0.001*^†^
4-year RFS	79	89	
5-year event	55	233	<0.001*^†^
5-year RFS	712	913	
**OS**			
3-year death	11	60	<0.001*^†^
3-year alive	447	478	
4-year death	8	17	0.060^†^
4-year alive	139	126	
5-year death	140	521	<0.001*^†^
5-year alive	2127	2125	

* Denotes statistical significance. †Denotes Fishers exact test. pCR, pathological complete response; RD, residual disease; EFS, event-free survival; RFS, recurrence-free survival; OS, overall survival.

### Pathological complete response and recurrence-free survival

**Fig. 4:**
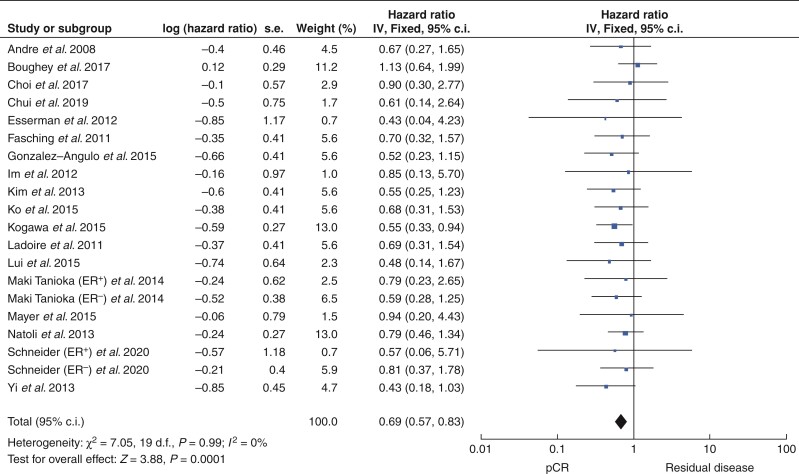
Forest plot comparing the hazards ratios relevant to recurrence-free survival for patients who successfully achieved pathological complete response following neoadjuvant therapies *versus* those with residual disease

Overall, 25 studies that included 4517 patients reported outcomes in relation to pCR as an indicator of RFS. Of these, 35.6 per cent successfully achieved pCR (1606 patients) *versus* 62.2 per cent with RD (2811 patients). pCR was associated with better RFS at 3 years, 4 years and 5 years after treatment (all *P* < 0.001) (*Table 1*). pCR predicted better RFS for patients with HER2^+^ breast cancer (HR 0.69, 95 per cent c.i. 0.57 to 0.83, *P* < 0.001, *I*^2^ = 0 per cent; 18 studies) (*Fig. 4* and *Fig. S7*). pCR also predicted better RFS after 3 years (HR 0.24, 95 per cent c.i. 0.12 to 0.49, *P* < 0.001, *I*^2^ = 0 per cent; 2 studies) (*Fig. S8*), after 4 years (HR 0.18, 95 per cent c.i. 0.05 to 0.70, *P* < 0.001, *I*^2^ = 0 per cent; 3 studies) (*Fig. S9*), and after 5 years (HR 0.28, 95 per cent c.i. 0.21 to 0.39, *P* < 0.001, *I*^2^ = 14 per cent; 8 studies) (*Fig. S10*).

### Pathological complete response and overall survival

**Fig. 5:**
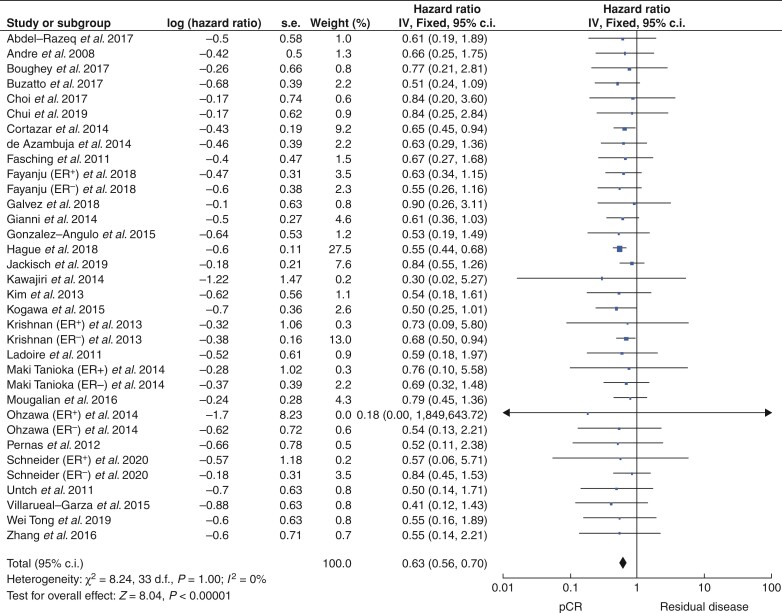
Forest plot comparing the hazards ratios relevant to overall survival for patients who successfully achieved pathological complete response following neoadjuvant therapies *versus* those with residual disease

**Fig. 6:**
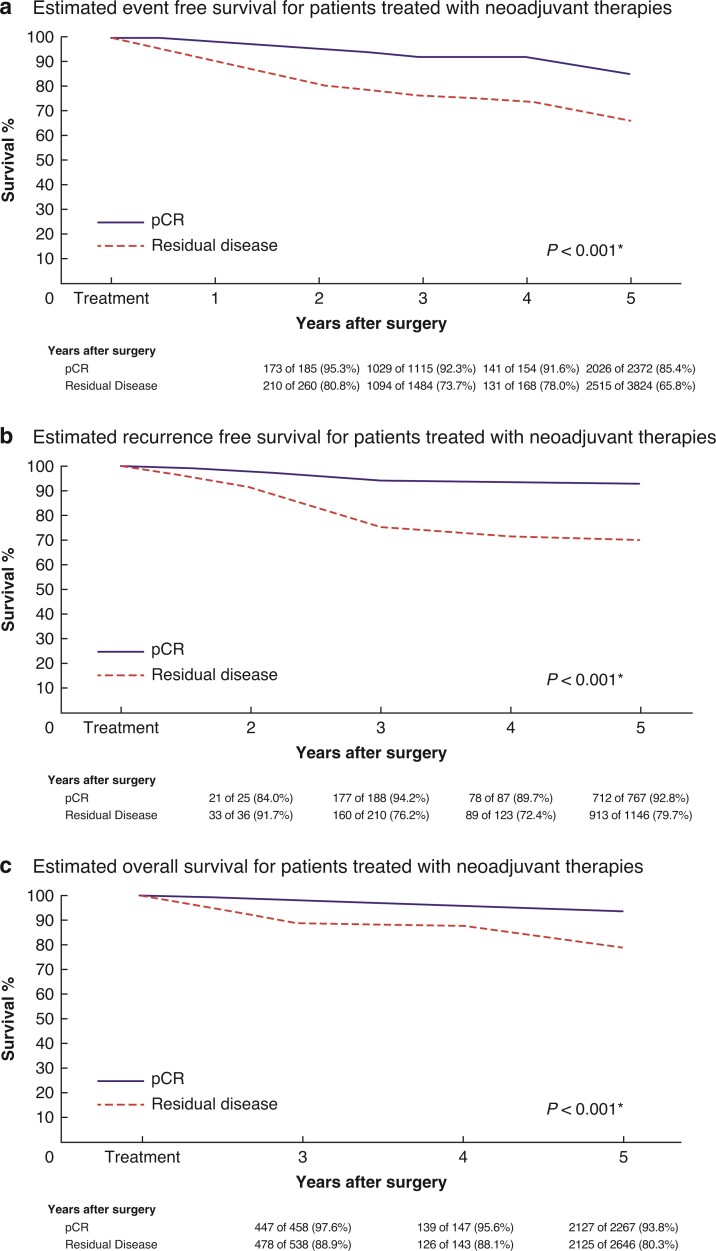
Estimated survival curves for (a) event-free, (b) recurrence-free, and (c) overall survival for patients achieving a pathological complete response to neoadjuvant therapies *versus* those with residual disease at the time of surgery

Overall, 32 studies that included 16 479 patients reported outcomes in relation to pCR as an indicator of OS (38.7 per cent successfully achieving pCR (6374 patients) *versus* 60.8 per cent with RD (10 020 patients). pCR was associated with better OS at 3 years and 5 years after treatment (both *P* < 0.001) (*Table 1*). pCR predicted better OS for patients with HER2^+^ breast cancer (HR 0.63, 95 per cent c.i. 0.56 to 0.70, *P* < 0.001, *I*^2^ = 0 per cent; 29 studies) (*Fig. 5* and *Fig. S11*). pCR also predicted better OS after 3 years (HR 0.25, 95 per cent c.i. 0.13 to 0.47, *P* < 0.001, *I*^2^ = 0 per cent; 5 studies) (*Fig. S12*), after 4 years (HR: 0.35, 95 per cent c.i. 0.12 to 1.02, *P* = 0.050, *I*^2^ = 0 per cent; 3 studies) (*Fig. S13*), and after 5 years (HR 0.26, 95 per cent c.i. 0.20 to 0.33, *P* < 0.001, *I*^2^ = 0 per cent; 10 studies) (*Fig. S14*). Estimated EFS, RFS, and OS curves for patients achieving a pCR *versus* those with RD are shown in *Fig. 6*.

## Discussion

This is the largest meta-analysis evaluating the role of pCR as a surrogate biomarker of survival for patients with overexpression of HER2 in their breast cancer. There are 78 studies encompassing more than 25 000 patients included in this analysis and the results highlight the importance of pCR as a positive prognostic biomarker for this patient group. These findings are consistent with the previous work of Broglio *et al*.^93^; however, this analysis provides additional data from 40 studies that were not previously included in their analysis. Furthermore, these results quantify the anticipated survival advantage for patients with HER2^+^ breast cancer who achieve pCR compared with their counterparts with RD at several annual timepoints after treatment of their cancer. This analysis highlights the value of successfully achieving pCR in the modern breast cancer treatment paradigm.

The present study is the first to evaluate the estimated survival advantage to achieving pCR specific to EFS, RFS, and OS in HER2^+^ breast cancer. In assessing pCR in relation to EFS, analysis was performed on 43 independent patient cohorts, which clearly illustrated pCR as an informative predictor of enhanced EFS (HR 0.67, 95 per cent c.i. 0.60 to 0.74). This illustrates the coherent message that pCR enhances clinical outcomes in HER2^+^ breast cancer, which is further reinforced by the enhanced outcomes at 2, 3, 4, 5, and 10 years. The impact of successfully achieving pCR on survival is evident as early as 2 years after treatment, and despite hazard ratios somewhat stabilizing at annual intervals from this point onwards, the estimated survival curves highlight the decline in anticipated survival for those with RD compared with those with pCR. This indicates that patients who achieve pCR are increasingly likely to be ‘cured’ of relapse from 2 years after resection, associating the greatest risk with the initial few months after treatment. Therefore, recurrence risk in those with pCR is most evident during the initial phase of remission, highlighting the appropriateness for close monitoring of these patients during this phase of remission. However, for those with RD, the risk of dissemination and recurrence seems to be spread more gradually across the years following completion of the adjuvant phase of their treatment, leading to their estimated EFS, RFS, and OS to be inferior to those achieving a pCR.

Despite these contrasting outcomes for those with pCR *versus* those with RD after receiving NAC, patients who successfully achieve pCR currently receive similar treatment regimens to those with RD^8^. Recently, von Minckwitz *et al*. successfully challenged this concept through the results of the KATHERINE study, which illustrated that patients with RD after receiving NAC who receive trastuzumab emtansine (T-DM1) outperform those treated with conventional trastuzumab (3-year disease-free survival for those receiving T-DM1 was 88.3 per cent *versus* 77.0 per cent for those receiving trastuzumab)^94^. This seminal study has facilitated a personalized approach to treating locally advanced HER2^+^ disease, while bringing into question the clinical validity of performing a second biopsy following neoadjuvant therapy to substratify patients into ‘complete responders’ and ‘non-responders’, which may guide decision-making in relation to further tailoring treatment strategies in accordance with the efficacy of initial NAC^95^. At present, the current molecular classification is based in principle upon practical, actionable biomarkers (namely the principal oestrogen and progesterone steroid hormone receptors, HER2 status, and Ki-67 proliferation indices), which guide therapeutic decision-making. Based on the results of this analysis, the unsuccessful ascertainment of pCR could provide an indication for prescribing further NAC or additional multimodal therapy in the adjuvant setting, should a tumour be identified on the second interval core biopsy. Therefore, histopathological confirmation of pCR after NAC seems a plausible means of patient substratification in those being treated with conventional NAC for HER2^+^ disease, once performed in a consistent and reproducible manner.

The seminal work of Cortazar *et al*. has illustrated the prognostic role of pCR with respect to EFS and OS^72^, and has been validated by Broglia *et al*. and Spring *et al*.^5,93^. Despite this, these authors failed to provide insight into the risk of index cancer recurrence following pCR. The current analysis is the first to assess this RFS as a primary outcome measure, with data from 20 independent cohorts estimating enhanced survival for those achieving pCR *versus* those with RD (HR 0.69, 95 per cent c.i. 0.57 to 0.83). Furthermore, 3-year, 4-year, and 5-year follow-ups suggest that recurrence rates are reduced in those with pCR. This is unsurprising when considering that surgical oncology relies on zero-order kinetics^96^; 100 per cent of excised tumour cells are killed with ‘clear’ margins of ‘normal’ breast parenchyma to ensure locoregional and disease control. Successful pCR involves complete eradication of cancer cells on the pathological specimen following neoadjuvant therapies^4^; this provides primary clearance of cancer from local host tissue, before surgeons perform a complete resection. Traditionally, tumour burden has been a useful biomarker of clinical prognostication of breast carcinoma^97^, with the concept of residual tumour burden (RTB) informing prognosis, risk of recurrence, and overall mortality for patients treated with neoadjuvant therapies for their breast neoplasms^98^. It is therefore theoretically intuitive that pCR, a compatible analogue of RTB after neoadjuvant therapies, is a sensitive, and informative surrogate to clinical outcomes in cancer.

This systematic review and meta-analysis is subject to a number of limitations. Various neoadjuvant therapeutic strategies have been evaluated in this study, some of which provide limited data within the context of current best practice guidelines. Moreover, the present analysis fails to make distinctions between pCR rates and survival based on steroid hormone receptor status; oestrogen receptor status is now embedded into the accepted molecular taxonomy of breast cancer, and routine measurement is critical for therapeutic decision making^99^. Additionally, this analysis fails to estimate survival based on stage-matched cohorts of patients who achieved pCR and RD (as outlined in *Table S2*), potentially limiting the conclusions that may be drawn. Similarly, surgical data, and details of adjuvant therapeutic strategies were not taken into consideration in this analysis. The authors also acknowledge that pCR may be considered a blunt instrument in providing accurate and informative estimations of survival in oncological practice. Despite these shortcomings, the authors wish to further emphasize the potential prognostic benefit of quantifying pCR as a biomarker of better clinico-oncological and survival outcomes in HER2^+^ breast cancer.

The present systematic review and meta-analysis highlights the prognostic significance of pCR as a surrogate biomarker to enhanced survival in HER2^+^ breast cancer. Achieving successful pCR to neoadjuvant therapies provides an anticipated survival advantage over patients with RD at annual time points following the treatment of their index cancer. Therefore, pCR should be perceived as an informative clinical parameter of prognosis. These results indicate that pCR should be included as a primary analytical endpoint in future trials evaluating the role of neoadjuvant therapies, with efforts focused around enhancing pCR rates and consequent enhancement of clinical outcomes by proxy.

## Supplementary Material

zrac028_Supplementary_Data

## Data Availability

Data will be made available upon reasonable request to the corresponding author.
