# Supplementary material for: Pathological complete response as a surrogate to improved survival in human epidermal growth factor receptor-2-positive breast cancer: systematic review and meta-analysis
Source: BJS Open. 2022 May 4;6(3):zrac028. doi: 10.1093/bjsopen/zrac028 (PMC9071230; doi:10.1093/bjsopen/zrac028)

**Pathological Complete Response as a Surrogate to Improved Survival in Human Epidermal Growth Factor Receptor-2 Breast Cancer: Systematic Review and Meta-Analysis**

**Supplementary Material.**

**Table S1.** Studies included in the current analysis with their neoadjuvant treatment regimens and definitions of pathological complete response.

| Study | Year | LOE | F/up  (Months) | N | N pCR | N Residual Disease | Treatment | Definition pCR |
| --- | --- | --- | --- | --- | --- | --- | --- | --- |
| Wang | 2016 | III | 36 | 549 | 237 | 312 | 4 cycles of PCH while 170 received PC | The absence of invasive carcinoma in both the breast tissue and lymph nodes of the resected specimen. Residual DCIS was included as pCR |
| Ingold Heppner | 2016 | I | 60 | 498 | 219 | 279 | 4 cycles of TEC +/- CP | The absence of invasive tumour cells in breast and lymph nodes (ypT0is/ypN0) |
| Ignatiadis | 2019 | I | 56 | 225 | 131 | 94 | Randomly assigned to H/PZ or with AC or AC-free regimen | ypT0/is ypN0 |
| Shiwei-Liu | 2020 | III | 44 | 187 | 124 | 63 | At least 4 cycles of H-containing NAT | No residual tumor cell in axillary lymph nodes |
| Boughey | 2017 | II | 49 | 204 | 103 | 101 | NAC and anti-HER2 therapy | No residual invasive disease in the breast or axillary lymph nodes. |
| Van Ramshorst | 2017 | III | 52 | 111 | 56 | 55 | Weekly paclitaxel PCH for 24 weeks and intermittent trastuzumab was administered without NAC | Absence of invasively growing tumour cells in both the breast and axillary lymph nodes at microscopic examination, irrespective of remaining *in situ* lesions (ypT0/is,ypN0) |
| Pivot | 2018 | I | 14 | 764 | 388 | 376 | NAC SB3 or TRZ for 8 cycles concurrently with chemotherapy | N/R |
| Zhang | 2017 | III | 21 | 119 | 15 | 104 | 1)TEC, 2)EC-T/P ± H, 3)TCH and 4) TC, NP, and NH | No residual invasive cancer cells in the primary tumor and regional lymph nodes (but some could be left over in in situ cancer) |
| Fayanju | 2018 | III | 35 | 5775 | 2625 | 3150 | Various strategies | ypT0 only |
| Mougalian | 2016 | III | 79 | 338 | 160 | 178 | Various strategies | Axillary nodal pCR |
| Jackisch | 2019 | I | 72 | 591 | 202 | 389 | Randomized to receive SC H (or IV H), administered with 4 cycles of TEC and 5-FU | Total pCR; absence of invasive neoplastic cells in the breast and ipsilateral axillary lymph nodes, regardless of ductal carcinoma in situ |
| Gianni | 2016 | I | 60 | 419 | 94 | 323 | Randomized to receive four cycles of 1)TH, 2)PZ and HT, 3)PZ and H, or 4)PZ and T | pCR in the breast |
| Swain | 2019 | I | 61 | 197 | 120 | 77 | TCH and L with weekly P | ypT0/Tis ypN0; absence of any invasive component in the resected breast specimen and absence of cancer on H&E evaluation of all resected lymph nodes |
| Haque | 2018 | III | 40 | 2274 | 880 | 1394 | Various strategies | ypT0N0 |
| Schneider | 2020 | II | 54 | 666 | 106 | 560 | NAC and H | The absence of residual invasive cancer cells in the breasts and axillary lymph nodes (ypT0/is + ypN0) |
| Gianni | 2014 | I | 65 | 235 | 68 | 167 | NAC +/- H | ypT0-is, pN0 |
| Sanchez-Munoz | 2013 | III | 81 | 39 | 13 | 26 | Various strategies | N/R |
| Steenbruggen | 2019 | III | 67 | 283 | 161 | 122 | Various strategies | ypT0/is, pN0 |
| Fujita | 2020 | III | 44 | 157 | 93 | 93 | Taxane-based regimens followed by AC-based regimens: 12 cycles of P or 4 cycles of T; and 4 cycles of FEC. | Absence of residual invasive cancer cells in the breast and axillary lymph nodes (ypT0/is + ypN0) |
| Ladoire | 2011 | III | 183 | 111 | 33 | 78 | AC‐based regimen or T + DC | Breast tissue without residual malignant epithelial invasive tumour, and associated with no microscopic evidence of tumour cells in axillary specimens |
| Zhang | 2016 | III | 47 | 137 | 66 | 71 | Various strategies | Overall pCR signifies the absence of invasive tumour cells in both the breast and the ALN, whereas axillary pCR refers to no malignancy in the ALN, no matter whether residual primary breast tumours are present or not. |
| Zhang | 2012 | N/R | N/R | 102 | 45 | 57 | N/R | N/R |
| Pierga | 2014 | II | 36 | 52 | 33 | 19 | N/R | Total or near total treatment effect with loss of nodal involvement |
| Hamy-Petit | 2015 | II | 33 | 287 | 112 | 175 | NAC regimens (AC-based or AC–taxane), with H | Absence of residual invasive cancer cells in the breast and axillary lymph nodes (ypT0/is+/ypN0). |
| Kim | 2013 | II | 63 | 229 | 114 | 115 | Various strategies | No residual invasive disease in the breast or axillary lymph nodes, regardless of non-invasive disease status. |
| Shinde | 2015 | II | 25 | 18 | 8 | 10 | 4 cycles of C and P with loading dose of H | No invasive disease in the breast and lymph nodes |
| Esserman | 2012 | I | 47 | 46 | 18 | 28 | CD as initial chemotherapy and H | Absence of invasive tumor in both breast and axillary lymph nodes after NAC |
| Untch | 2011 | N/R | N/R | 217 | 85 | 132 | N/R | N/R |
| Maki Tanioka | 2014 | III | 55 | 366 | 137 | 229 | Neoadjuvant AC and/or taxane - H with taxanes | pCR was defined as ypT0/is ypN0 |
| Spring | 2017 | III | 62 | 55 | 26 | 29 | NAC plus H | No residual invasive disease in the breast and axilla, with non-invasive residuals permitted, including ductal carcinoma in situ (ypT0/is ypN0) |
| Pernas | 2012 | II | 50 | 83 | 51 | 32 | P for 12 weeks followed by 4 cycles of FEC with H | No invasive residual carcinoma in both the breast and lymph nodes, regardless of the presence of ductal carcinoma in situ (ypT0/is ypN0) |
| Villarueal-Garza | 2015 | III | 47 | 244 | 119 | 125 | Taxanes and AC plus H | Complete absence of any residual invasive and in situ cancer in the breast and absence of any metastatic cells in the regional lymph nodes (ypT0, ypN0) |
| Den Brok | 2016 | III | 77 | 122 | 59 | 63 | N/R | N/R |
| Liu | 2015 | III | 33 | 116 | 42 | 74 | At least four cycles of H-based NAC | Absence of residual invasive tumor cells in the breast and axillary lymph nodes (ypT0/is + ypN0) |
| Hurvitz | 2019 | I | 37 | 418 | 223 | 195 | NAC; T plus PZ with NAC; DC plus dual anti-HER2 blockade (H and PZ) | ypT0/is, ypN0 |
| Buzatto | 2017 | III | N/R | 86 | 31 | 55 | 1) EC-T± H, 2) T+H, 3) FEC, 4) T+E and all patients completed 1 year of treatment with adjuvant H | Absence of residual invasive cancer in breast and lymph nodes |
| Schmidt | 2021 | III | 30 | 175 | 110 | 65 | H | N/R |
| Frayser Symmans | 2017 | III | N/R | 306 | 113 | 193 | P followed by combined FDC | N/R |
| Giacchetti | 2017 | I | 94 | 120 | 27 | 92 | 4 cycles of TEC +/- H | Absence of infiltrating tumour in breast and lymph nodes |
| Shimizu | 2009 | III | N/R | 125 | 43 | 82 | AC plus a taxane | Complete absence of residual invasive tumor cells in the primary tumor |
| Mayer | 2015 | III | 25 | 80 | 14 | 66 | C+P + loading H followed by infusion while receiving NCT | No invasive disease in the breast and lymph nodes |
| Schneeweiss | 2018 | I | 61 | 208 | 128 | 80 | 1) H+PZ+FEC, 2) H+PZ+D, or 3) C+H+PZ+D | Absence of invasive neoplastic cells during microscopic assessment of the primary tumour (bpCR; ypT0/is) or in the breast and axilla (tpCR; ypT0/Tis, ypN0). |
| de Azambuja | 2014 | I | 45 | 411 | 137 | 274 | L + IV H followed by an additional 12 weeks of H + P | ypT0 |
| Wei Tong | 2019 | III | 29 | 101 | 29 | 71 | AC + taxane or non AC + taxane regimen (EC-T, EC-P, TC, and PC) | No residual invasive carcinoma in breast and axilla |
| Zelnak | 2015 | I | 52 | 27 | 13 | 14 | 4 cycles of NP followed by V for 12 weeks with concurrent H | Absence of invasive disease in the breast and axilla |
| Kogawa | 2015 | III | 41 | 555 | 199 | 356 | NAC + H | No invasive disease in the breast and no tumor in the ipsilateral axillary lymph nodes |
| Wang | 2014 | II | 60 | 80 | 22 | 58 | CE-T or TE regimen | No invasive components in pathological specimens obtained through modified radical mastectomy after NAC |
| Yao | 2011 | III | 28 | 116 | 17 | 99 | 4-8 cycles of AC-based regimens | No invasive breast tumor cells in breast after NAC |
| Tulbah | 2002 | III | 25 | 52 | 13 | 39 | PC | N/R |
| Abdel-Razeq | 2017 | III | N/R | 121 | 32 | 87 | 4 cycles of AC-T | No evidence of invasive carcinoma in the breast |
| Choi | 2017 | III | 36 | 98 | 25 | 73 | 4-6 cycles of AC-T + H | ypT0/is ypN0 |
| Kurozumi | 2015 | III | 59 | 129 | 84 | 45 | 12 cycles of P or 4 cycles of D by 4 cycles of FEC. All patients also received H | Various definitions |
| Bayraktar | 2011 | III | 27 | 300 | 163 | 137 | P and H and FEC+H or TCH | Absence of invasive disease in the breast and the absence of micro‐ or macrometastases in the ipsilateral axillary lymph nodes |
| Zhang | 2020 | III | 33 | 234 | 49 | 185 | EC-PH or EC-P NAC | Absence of invasive residual breast tumor or invasive lesion in ipsilateral axillary nodes (ypT0 ypN0) |
| Gonzalez-Angulo | 2015 | III | 45 | 589 | 203 | 386 | P and H and FEC+H | No evidence of invasive cancer in the breast and lymph nodes (yT0/is,N0) |
| Galvez | 2018 | III | 61.2 | 77 | 10 | 67 | 4 cycles of DC +/- 12 weekly P | Absence of invasive cancer in the breast and axillary nodes, irrespective of carcinoma *in situ* (ypT0/is ypN0) |
| Chui | 2019 | N/R | N/R | 226 | 64 | 162 | 1)NAC, 2)NAC+H or 3)NAC + double H | N/R |
| Ohzawa | 2014 | III | 29 | 47 | 16 | 31 | AC-based regimens used: 1) FEC, 2) AC, and 3) EC. Taxane-based regimens were used: 1) T, 2) 4 cycles of T 3) P | No pathological evidence of residual invasive cancer in the breast, irrespective of the remaining intraductal components in the breast and axillary tumors |
| Cortazar | 2014 | I | 64 | 1989 | 586 | 1403 | Various | pT0/is ypN0 or ypT0 ypN0 |
| Saracchini | 2013 | II | 57 | 39 | 19 | 20 | 4 cycles of Dx + C followed by 12 cycles of T +H | Absence of residual invasive cancer in the breast and regional lymph nodes determined with standard histologic procedure, also in presence of residual in situ carcinoma |
| Andre | 2008 | III | 31 | 105 | 35 | 70 | P + FAC | No invasive cancer in the breast or in the lymph nodes |
| Gonzalez-Angulo | 2004 | III | 61 | 19 | 5 | 14 | 4 cycles of P or T | Absence of invasive disease in both the breast and the axillary lymph nodes at the time of definitive surgery |
| Le Tourneau | 2012 | III | 121 | 11 | 0 | 11 | 4 cycles of NAC with high-dose FAC (F, Dx, C) | No residual tumour cells in the breast tumour and lymph nodes in the axilla |
| Kawajiri | 2014 | III | 53 | 33 | 12 | 21 | FEC+ H | No evidence of residual invasive cancer in the breasts and lymph nodes, including in patients with non-invasive or *in situ* cancer and in patients whom no residual cancer cells were identified |
| Takada | 2014 | III | 42 | 776 | 399 | 377 | NAC containing H | Absence of residual invasive cancer cells in the breast and axillary lymph nodes (ypT0/is ypN0) |
| Fasching | 2011 | III | 34 | 92 | 37 | 55 | Various strategies | ypT0 ypN0 (no in situ allowed) |
| Guarneri | 2013 | N/R | N/R | 102 | 41 | 61 | AC + taxane | Complete disappearance of invasive carcinoma in breast and axillary lymph nodes |
| Natoli | 2013 | III | 32 | 205 | 96 | 109 | Various NAC + H | Absence of invasive and noninvasive breast cancer in the breast and axillary lymph nodes (ypT0 and ypN0, later on referred to as pCR/0) or the absence of invasive breast cancer in the breast and axillary lymph nodes (ypT0/is and ypN0, later on referred to as pCR/is) |
| Ju | 2013 | III | 47 | 53 | 53 | 0 | NAC +/- H | No residual invasive tumor in the breast surgical specimen removed following neoadjuvant therapy; patients with residual carcinoma in situ were considered to have a pCR |
| Gropper | 2011 | I | 100 | 78 | 15 | 63 | 1)PH with optional adjuvant C, or 2)NH with optional adjuvant C+/-H | Absence of invasive disease in breast and lymph nodes at surgery |
| Melichar | 2012 | III | 68 | 89 | 25 | 64 | Dx + P or Dx + C + P. | Complete disappearance of tumor cells in the operative specimen obtained at definitive surgery after neoadjuvant chemotherapy |
| Yi | 2013 | III | 47 | 76 | 18 | 58 | NAC | Absence of invasive tumor cells in the primary tumor sites (ductal carcinoma in situ may be present) |
| Krishnan | 2013 | III | 49 | 102 | 22 | 80 | FEC, AC, TE, FEC-T, AC-T or TE | Absence of invasive carcinoma in both breast and axillary lymph nodes in the post-surgery specimen |
| Hurley | 2006 | II | 43 | 48 | 11 | 37 | TC + H with FL, followed by surgery, adjuvant Dx + C | Disappearance of invasive disease in breast by pathologic examination, included specimens containing only residual ductal carcinoma-in-situ; pCR in axilla was absence of positive lymph nodes by hematoxylin and eosin staining. |
| Im | 2012 | I | 40 | 53 | 17 | 36 | P and G followed by H | Complete absence of viable invasive tumor cells on pathologic examination, regardless situ cancer |
| Ko | 2015 | III | 55 | 59 | 22 | 37 | 1)Ad-C, 2)Ad-T, 3)AC-T +/- H or P, 4)AC-T with H, 5)PT, 6) HT, or 7)PH | ypT0/is ypN0 |
| Lui | 2015 | III | 32 | 108 | 41 | 67 | 4 cycles H-based NAT | ypT0/is+ypN0 |
| Villarueal-Garza | 2016 | III | 51 | 430 | 193 | 237 | NAC | No invasive residual tumor in the breast or lymph nodes [noninvasive breast residuals allowed (ypT0/is, ypN0)]. |

LOE; level of evidence, F/Up; Follow up, N; number, pCR; pathological complete response, PCH; paclitaxel, carboplatin, and trastuzumab, PC; paclitaxel and carboplatin, DCIS; ductal carcinoma in-situ, NAC; neoadjuvant chemotherapy, HER2; human epidermal growth factor receptor-2, FEC; 5-fluorouracil, epirubicin and cyclophosphamide, TEC; docetaxel plus epirubicin plus cyclophosphamide, EC-T/P ± H; epirubicin and cyclophosphamide sequential docetaxel or paclitaxel with or without trastuzumab, CP; capecitabine, TCH; docetaxel plus carboplatin plus trastuzumab, P; Paclitaxel, T; docetaxel , TC; docetaxel plus cyclophosphamide, NP; vinorelbine plus cisplatin, NH; vinorelbine plus trastuzumab, H; trastuzumab, 5-FU; 5-fluorouracil, PZ; pertuzumab, L; lapatinib, AC; anthracycline, NAT; neoadjuvant therapies, SB3; a trastuzumab similar, SC; subcutaneous, IV; intravenous, HT; trastuzumab and docetaxel, ALN; axillary lymph node, E; epirubicin, EC; epirubicin and cyclophosphamide, Dx; doxorubicin, FAC; 5-fluorouracil, anthracycline and cyclophosphamide, FEC-T; 5-fluorouracil, epirubicin, cyclophosphamide and docetaxel, AC-T; anthracycline and docetaxel, TE; docetaxel and epirubicin, FL; filgrastim, G; gemcitabine, Ad-C; adriamycin with cyclophosphamide, Ad-T; adriamycin with docetaxel, AdC-T; adriamycin with cyclophosphamide plus docetaxel, PT; pertuzumab and docetaxel

**Table S2.** Clinicopathological data and correlation with achieving a pathological complete response to neoadjuvant therapies.

| Parameter | pCR | RD | P-value |
| --- | --- | --- | --- |
| HER2+  LBBC | 713  487 | 854  1089 | <0.001*† |
| T1/2  T3/4 | 272  203 | 315  645 | <0.001*† |
| T1  T2  T3  T4 | 170  1627  404  246 | 657  5927  2301  1167 | <0.001*χ² |
| N0  N+ | 1285  1247 | 5283  5244 | 0.611† |
| N0  N1  N2  N3 | 1285  152  61  46 | 5283  159  90  88 | <0.001*χ² |
| Grade 1/2  Grade 3 | 792  1086 | 4655  2797 | <0.001*† |
| Grade 1  Grade 2  Grade 3 | 33  606  1086 | 293  4010  2797 | <0.001*χ² |
| LVI present  LVI absent | 14  32 | 100  82 | 0.005† |
| ER+  ER- | 191  553 | 296  377 | <0.001*† |
| PgR+  PgR- | 133  277 | 382  427 | 0.001*† |
| Ki-67 <14%  Ki-67 >14% | 15  86 | 46  130 | 0.004*† |

pCR; pathological complete response, RD; residual disease, HER2; human epidermal growth factor receptor 2, LBBC; luminal B breast cancer, T; tumour stage, N; nodal stage, N+; node positive, LVI; lymphovascular invasion, ER; estrogen receptor, PgR; progesterone receptor

* denotes statistical significance

† denotes Fishers Exact test

χ² denotes Chi-Square test

**Figure S1.** Funnel plot comparing event free survival for patients who successfully achieved pathological complete response following neoadjuvant therapies versus those with residual disease.


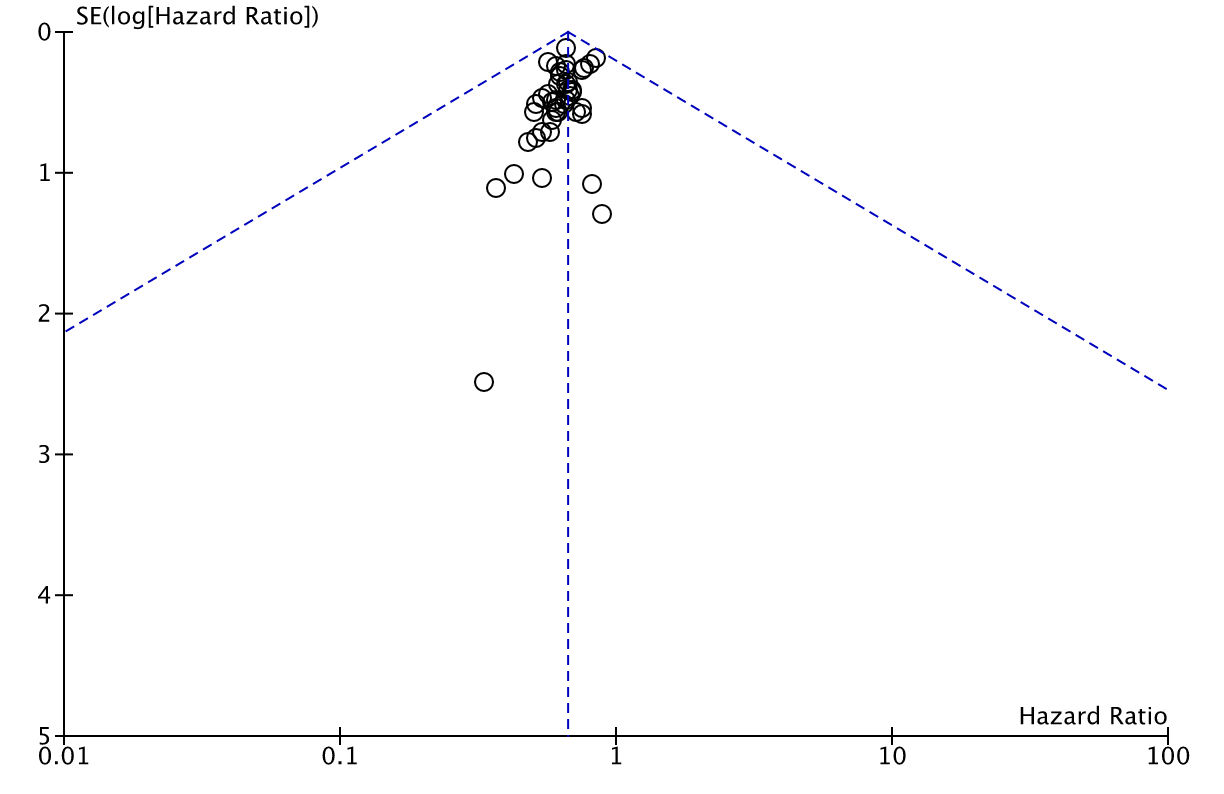


**Figure S2.** Forest and associated funnel plots comparing 2-year event free survival for patients who successfully achieved pathological complete response following neoadjuvant therapies versus those with residual disease.


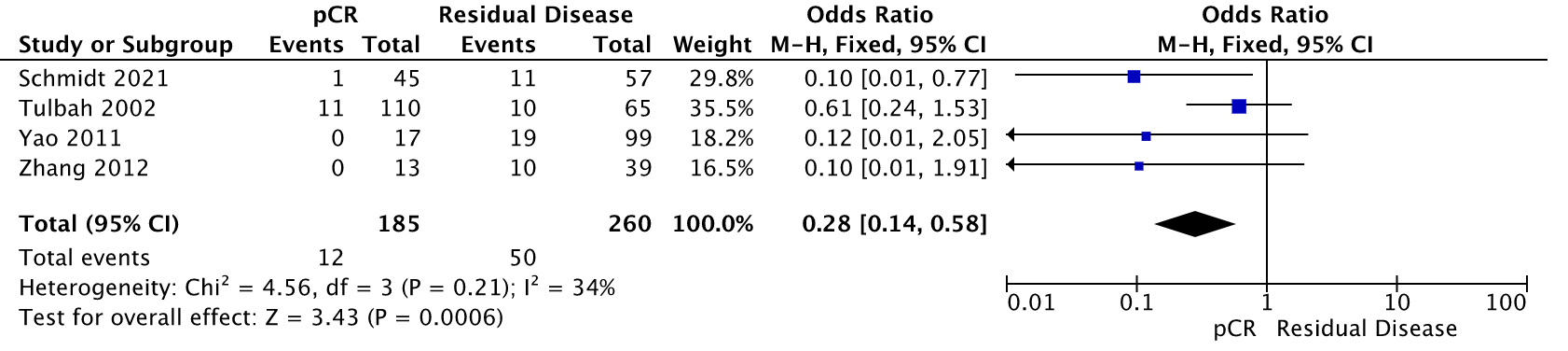


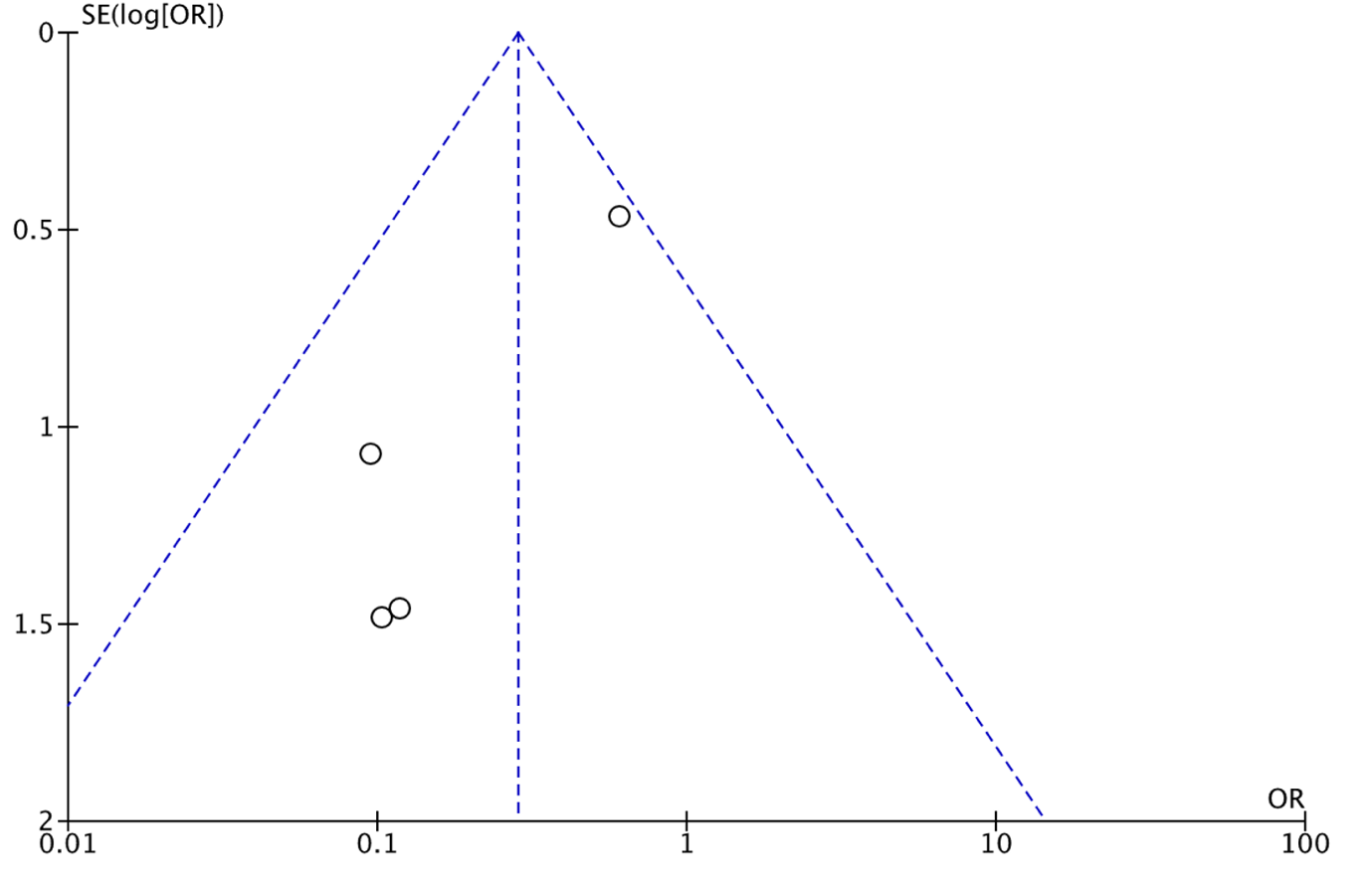


**Figure S3.** Forest and associated funnel plots comparing 3-year event free survival for patients who successfully achieved pathological complete response following neoadjuvant therapies versus those with residual disease.

**
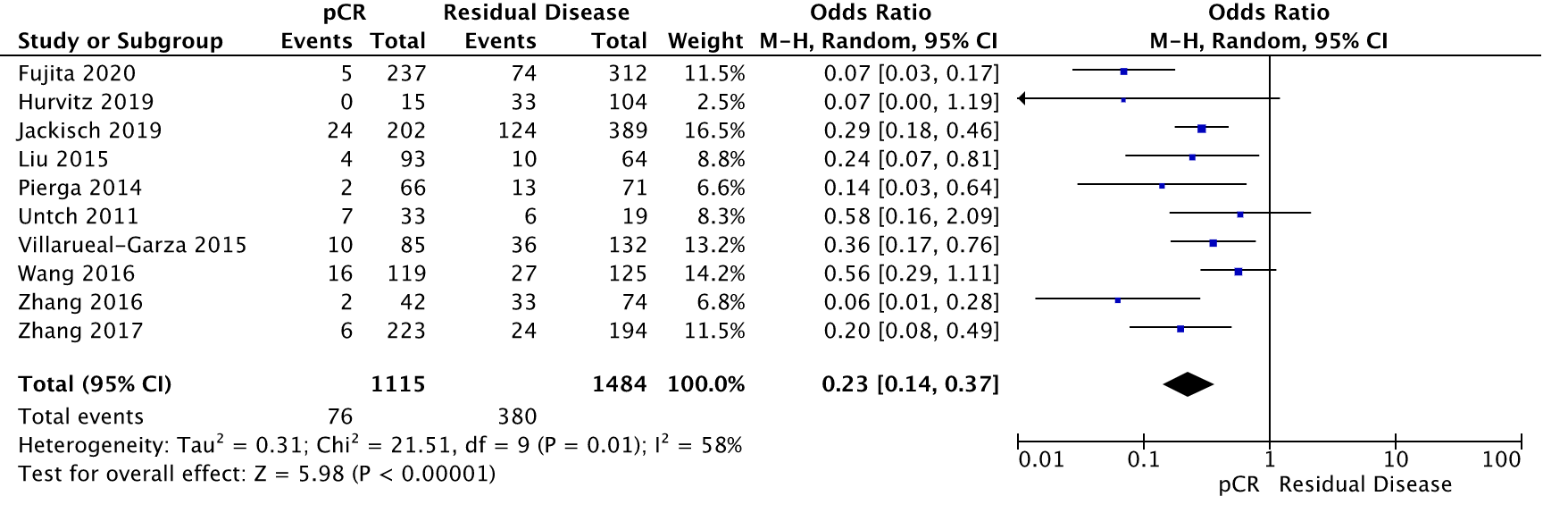

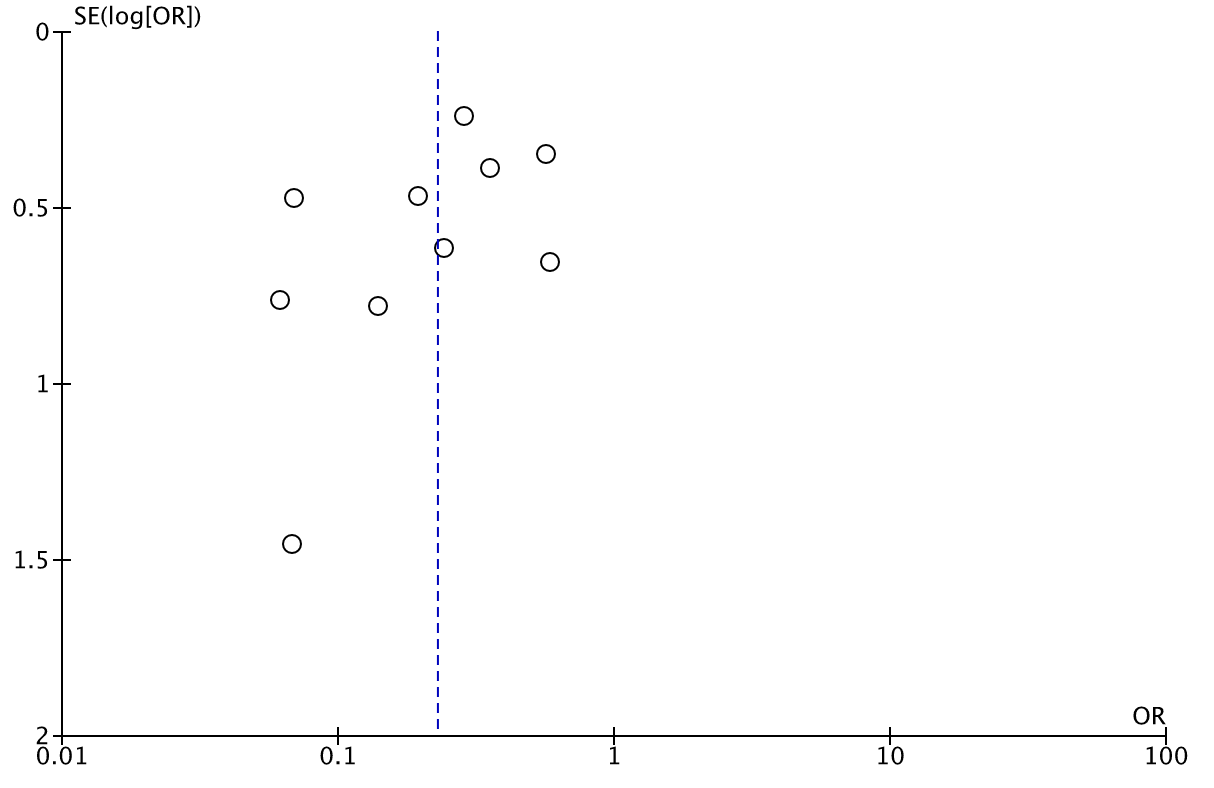
**

**Figure S4.** Forest and associated funnel plots comparing 4-year event free survival for patients who successfully achieved pathological complete response following neoadjuvant therapies versus those with residual disease.


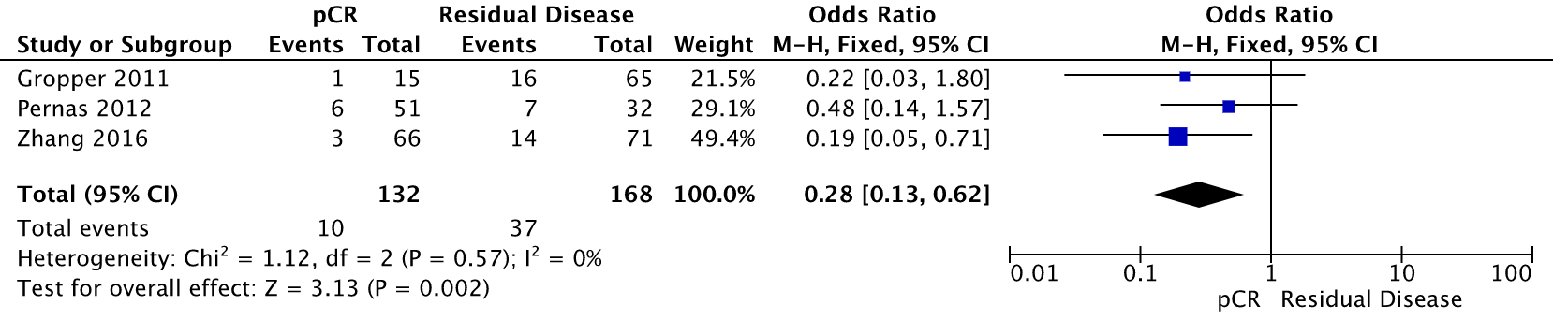

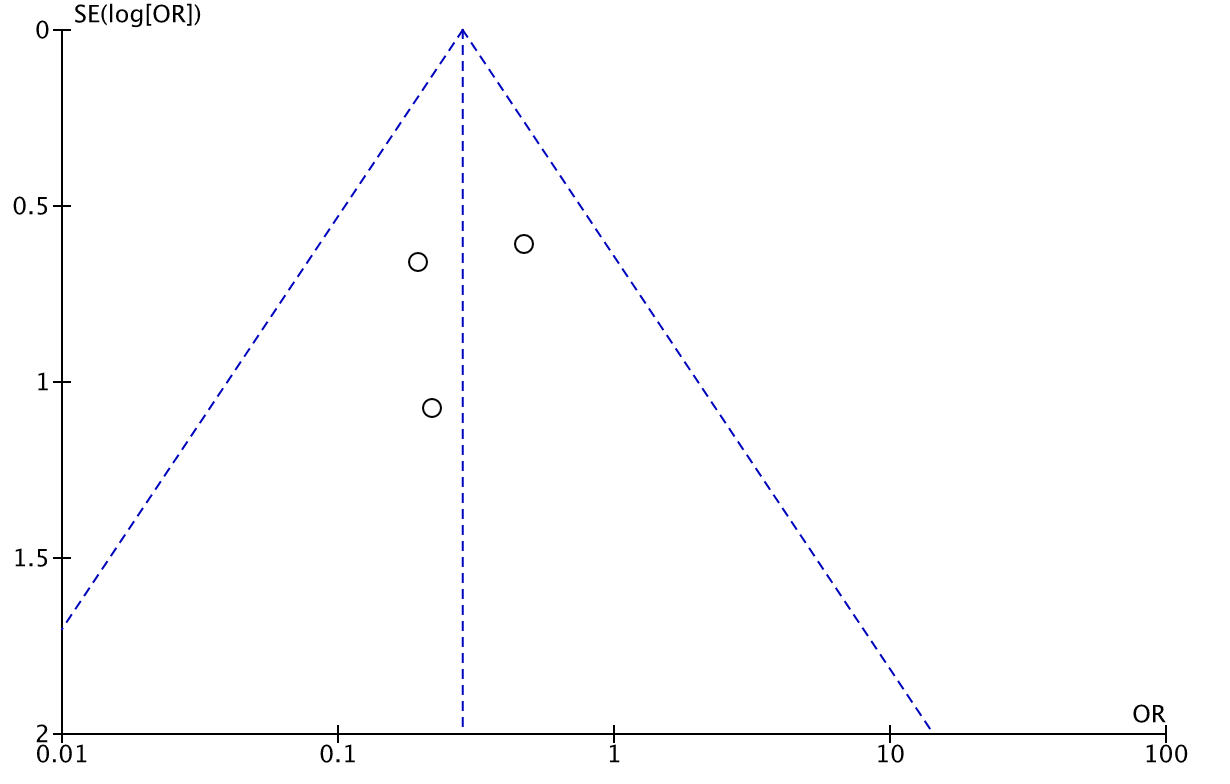


**Figure S5.** Funnel plot comparing 5-year event free survival for patients who successfully achieved pathological complete response following neoadjuvant therapies versus those with residual disease.


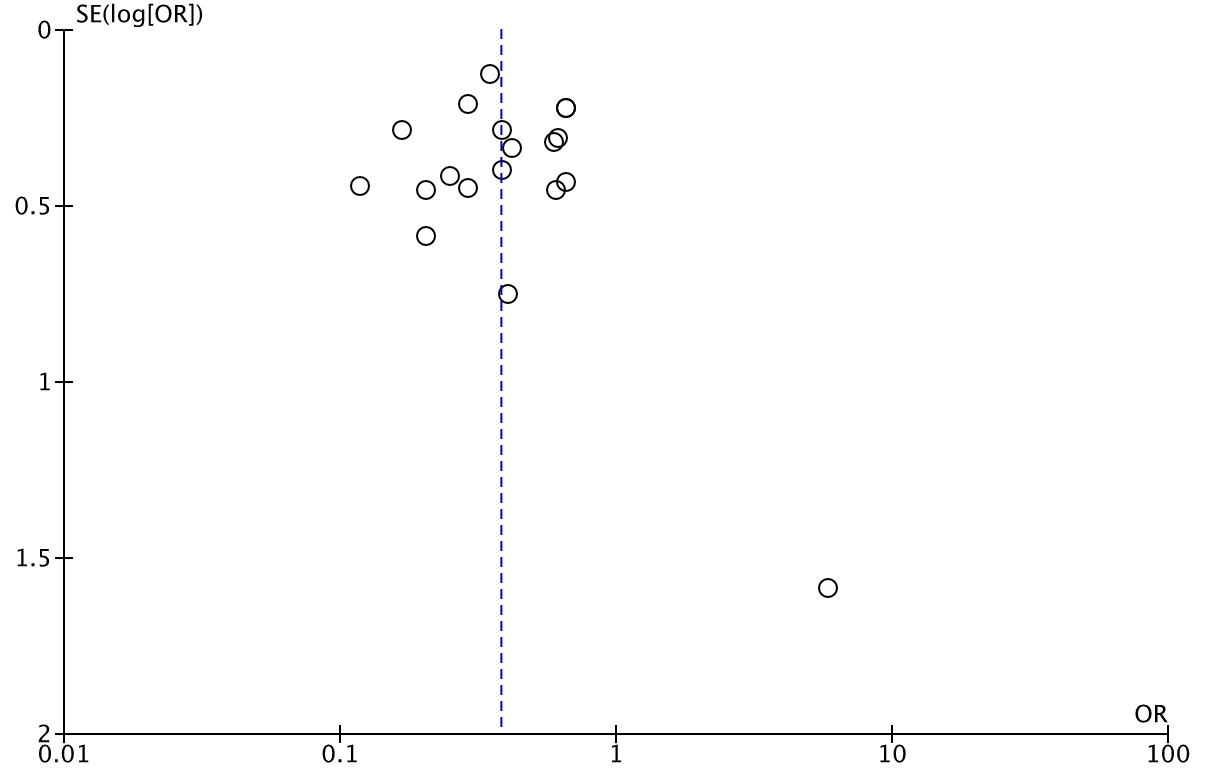


**Figure S6.** Forest and associated funnel plots comparing 10-year event free survival for patients who successfully achieved pathological complete response following neoadjuvant therapies versus those with residual disease.


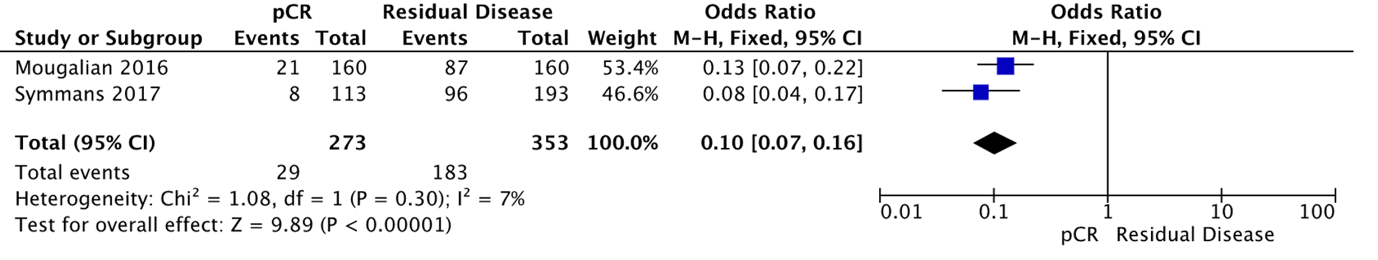


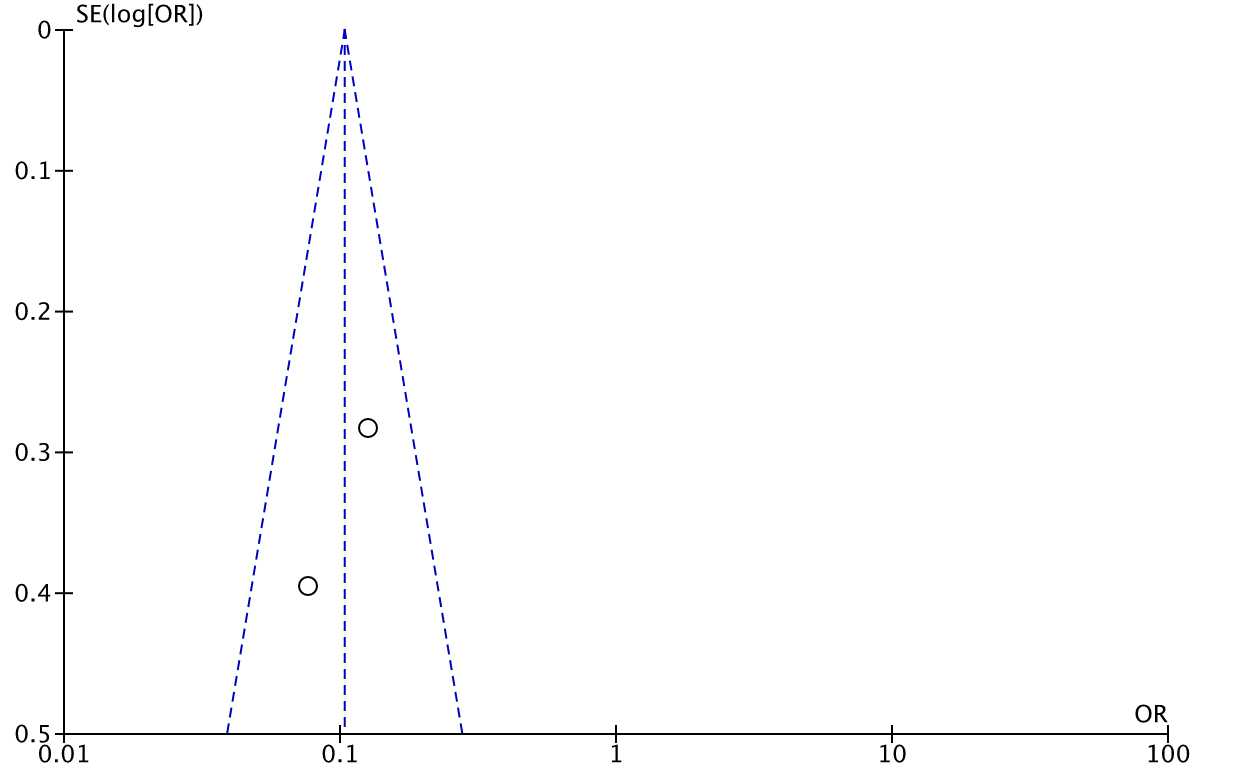


**Figure S7.** Funnel plot comparing recurrence-free survival for patients who successfully achieved pathological complete response following neoadjuvant therapies versus those with residual disease.


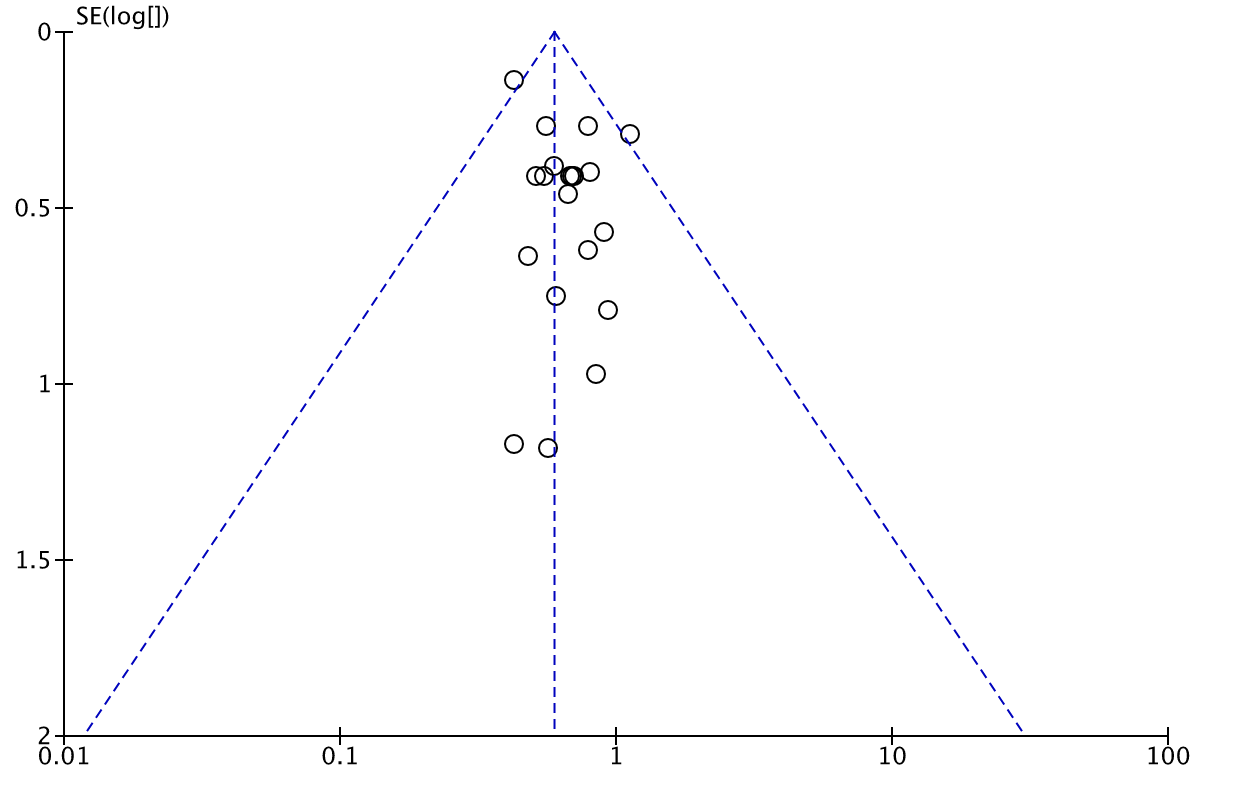


**Figure S8.** Forest and associated funnel plots comparing 3-year recurrence-free survival for patients who successfully achieved pathological complete response following neoadjuvant therapies versus those with residual disease.


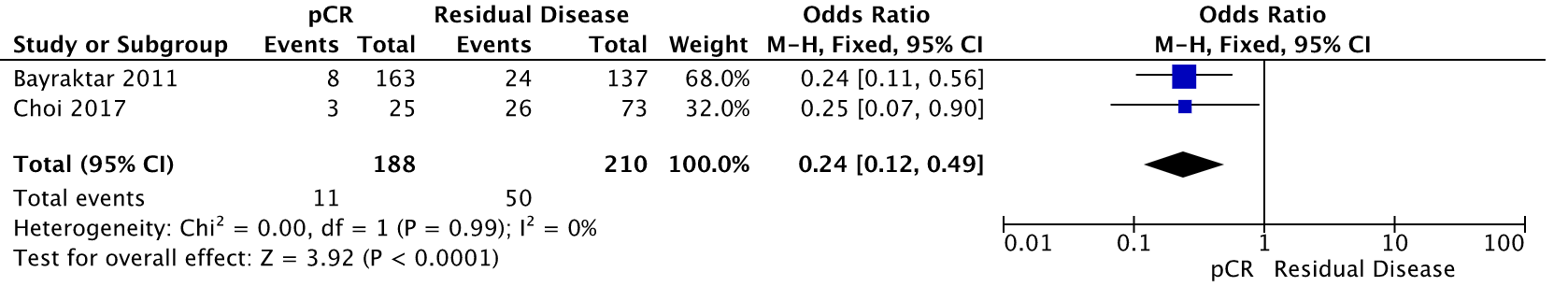

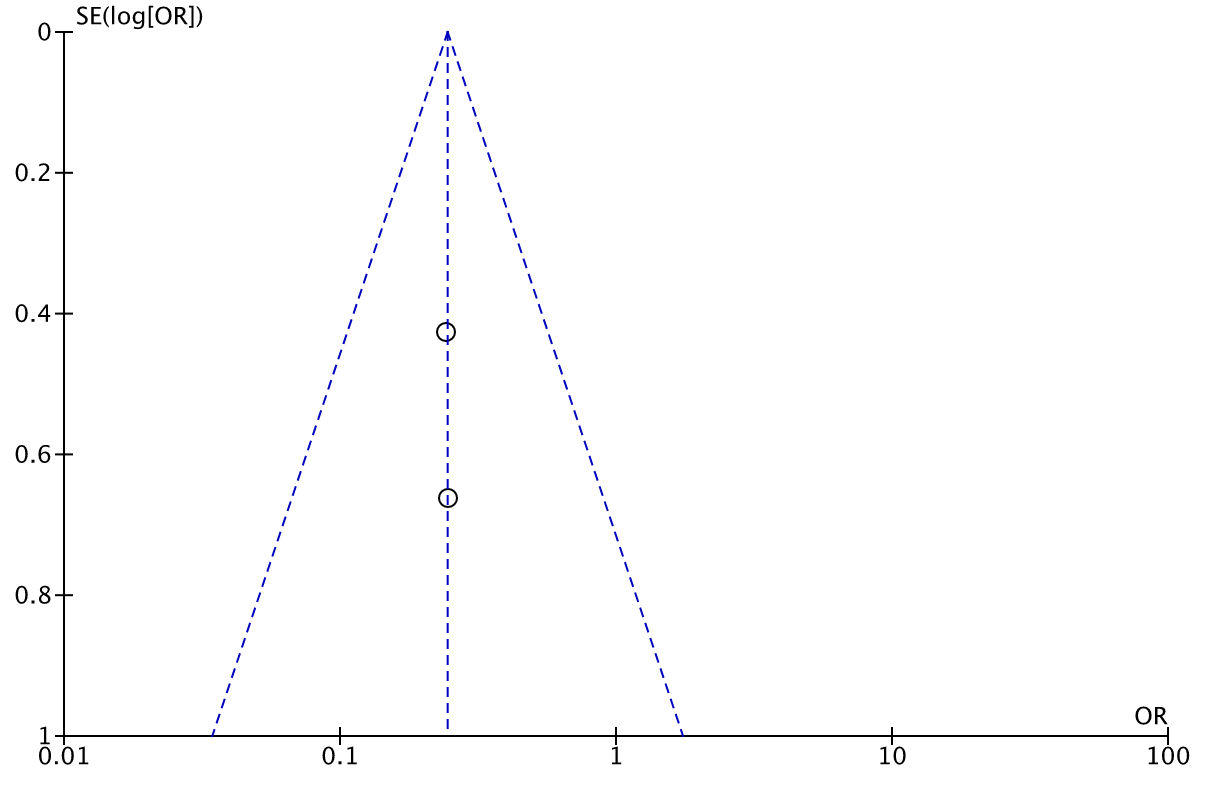


**Figure S9.** Forest and associated funnel plots comparing 4-year recurrence-free survival for patients who successfully achieved pathological complete response following neoadjuvant therapies versus those with residual disease.


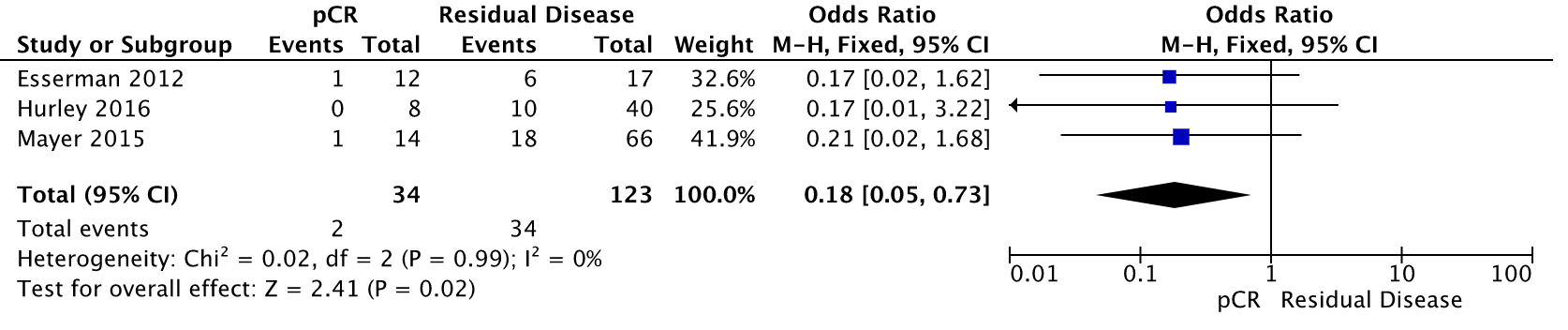

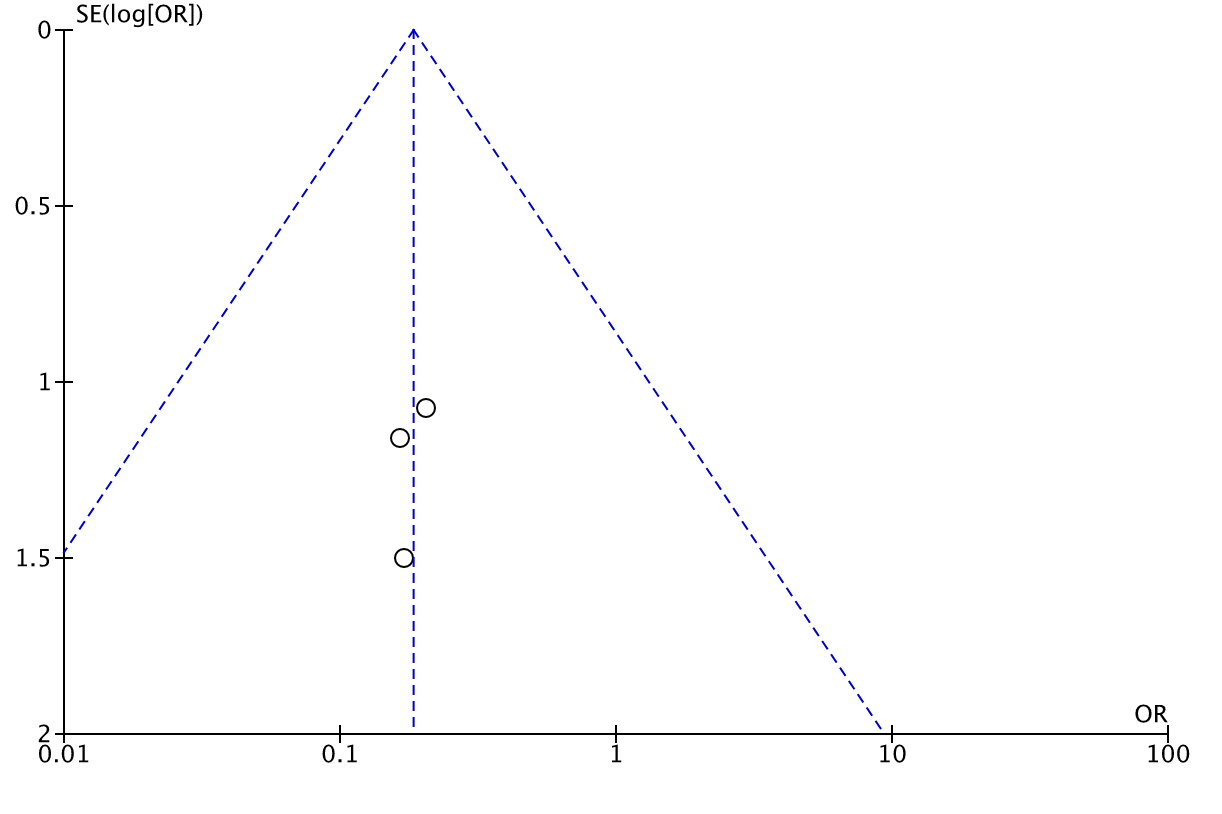


**Figure S10.** Forest and associated funnel plots comparing 5-year recurrence-free survival for patients who successfully achieved pathological complete response following neoadjuvant therapies versus those with residual disease.


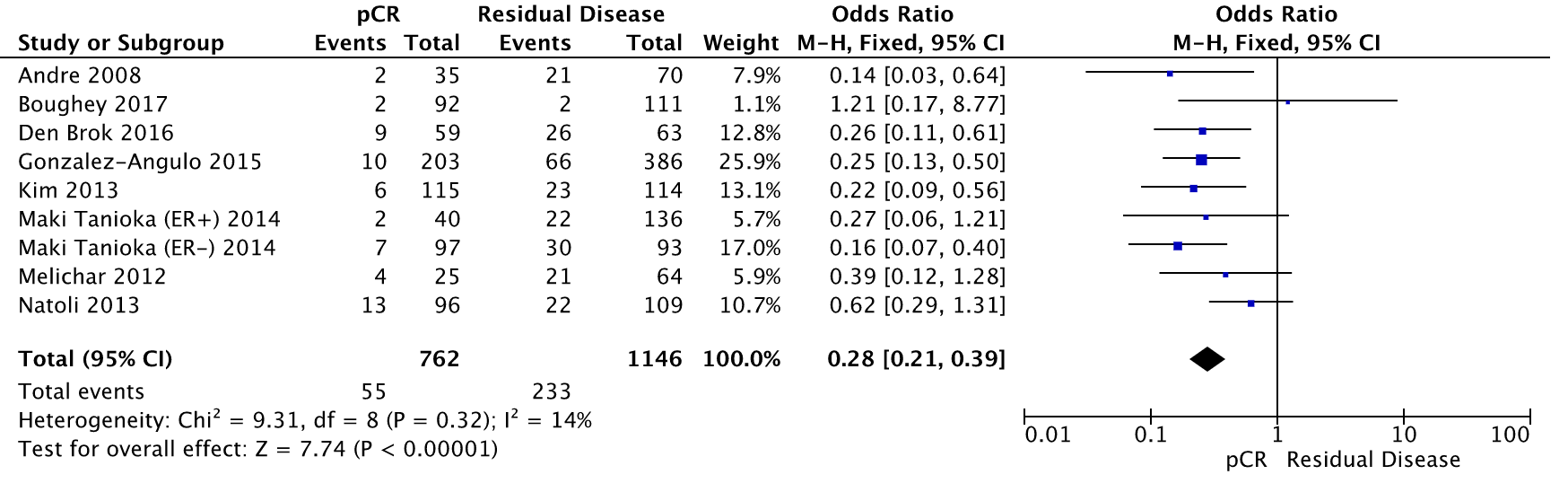

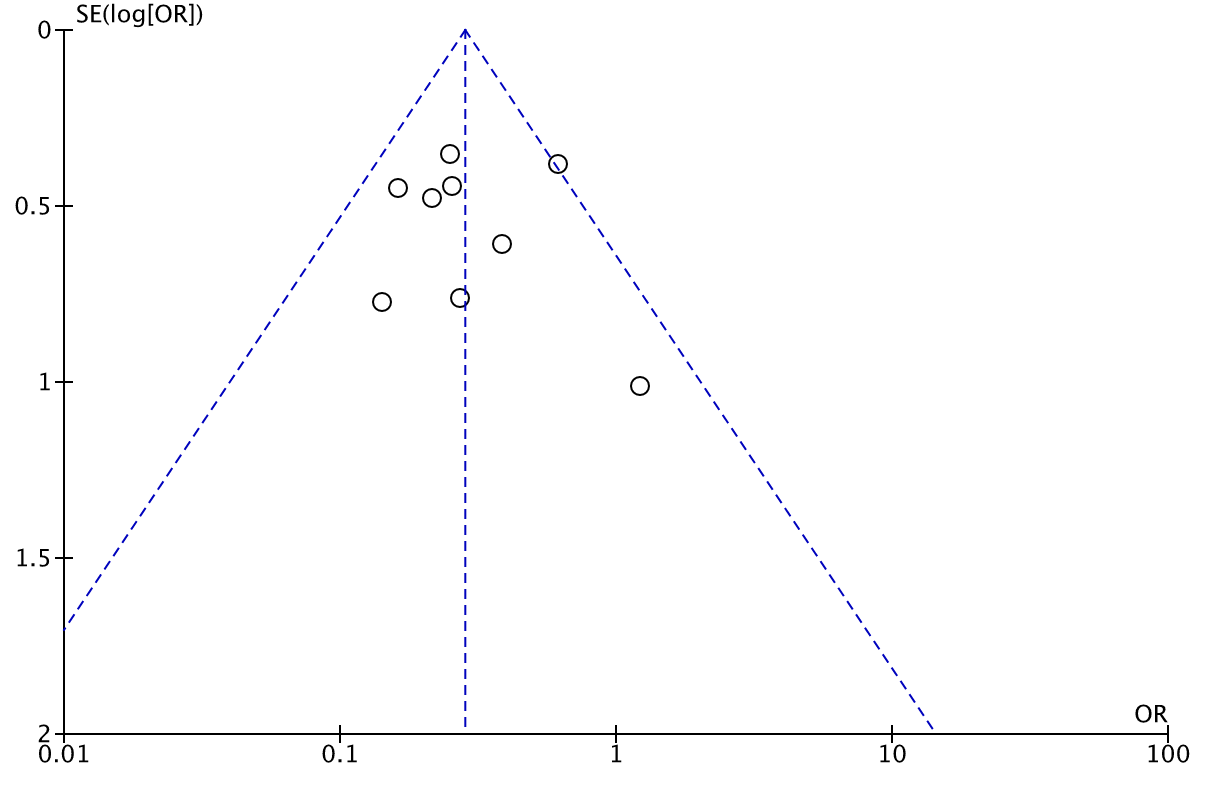


**Figure S11.** Funnel plot comparing overall survival for patients who successfully achieved pathological complete response following neoadjuvant therapies versus those with residual disease.


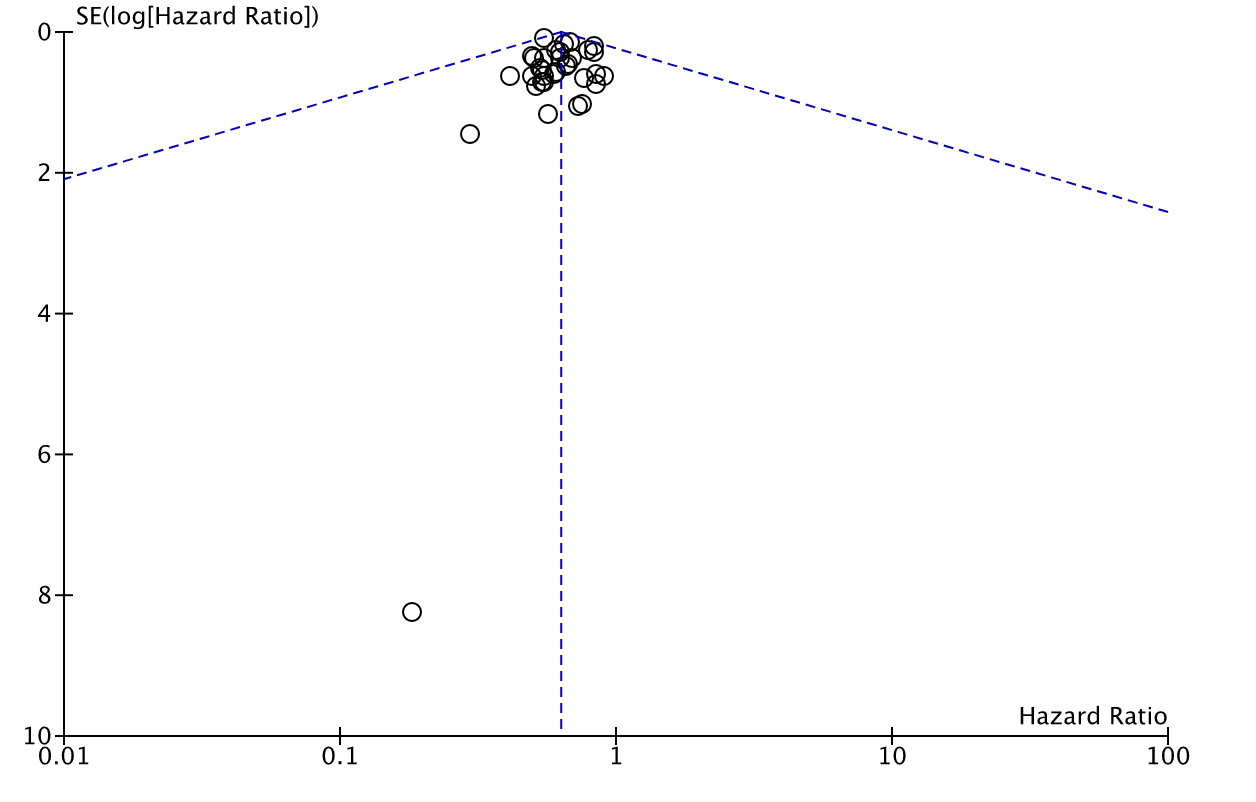


**Figure S12.** Forest and associated funnel plots comparing 3-year overall survival for patients who successfully achieved pathological complete response following neoadjuvant therapies versus those with residual disease.


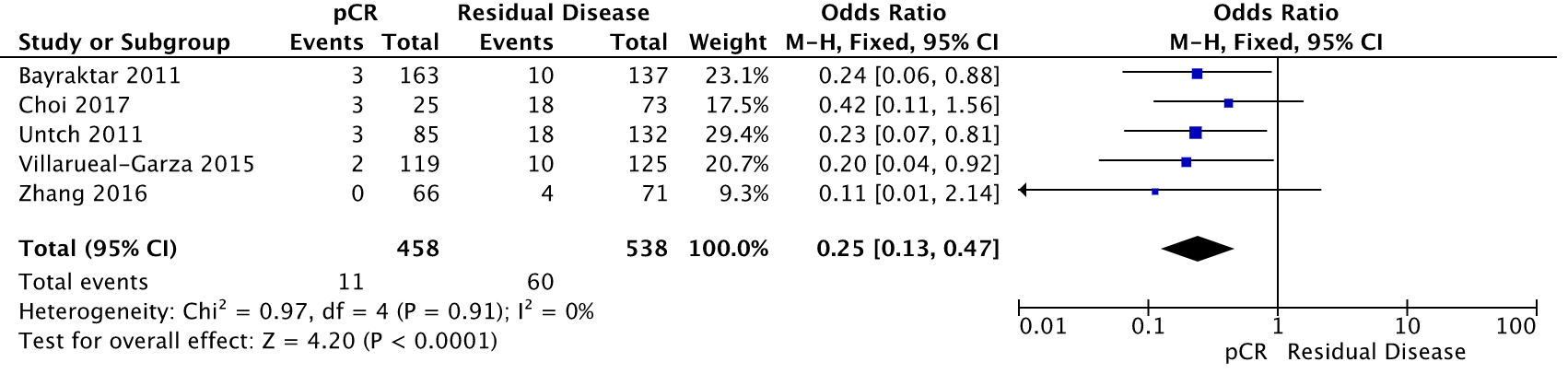

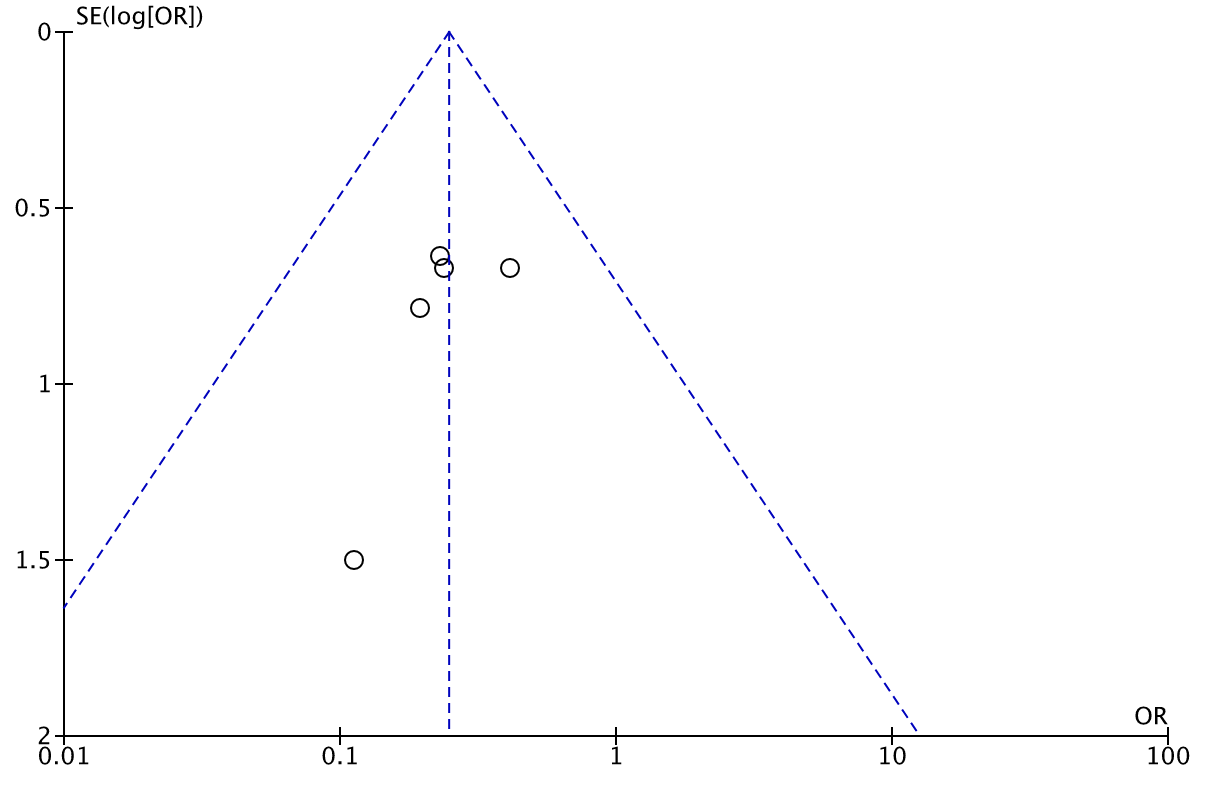


**Figure S13.** Forest and associated funnel plots comparing 4-year overall survival for patients who successfully achieved pathological complete response following neoadjuvant therapies versus those with residual disease.


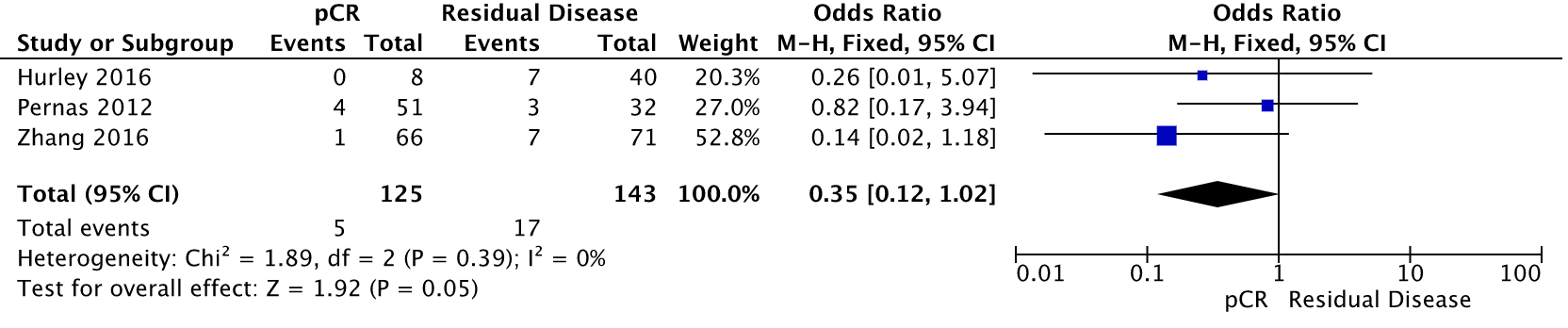

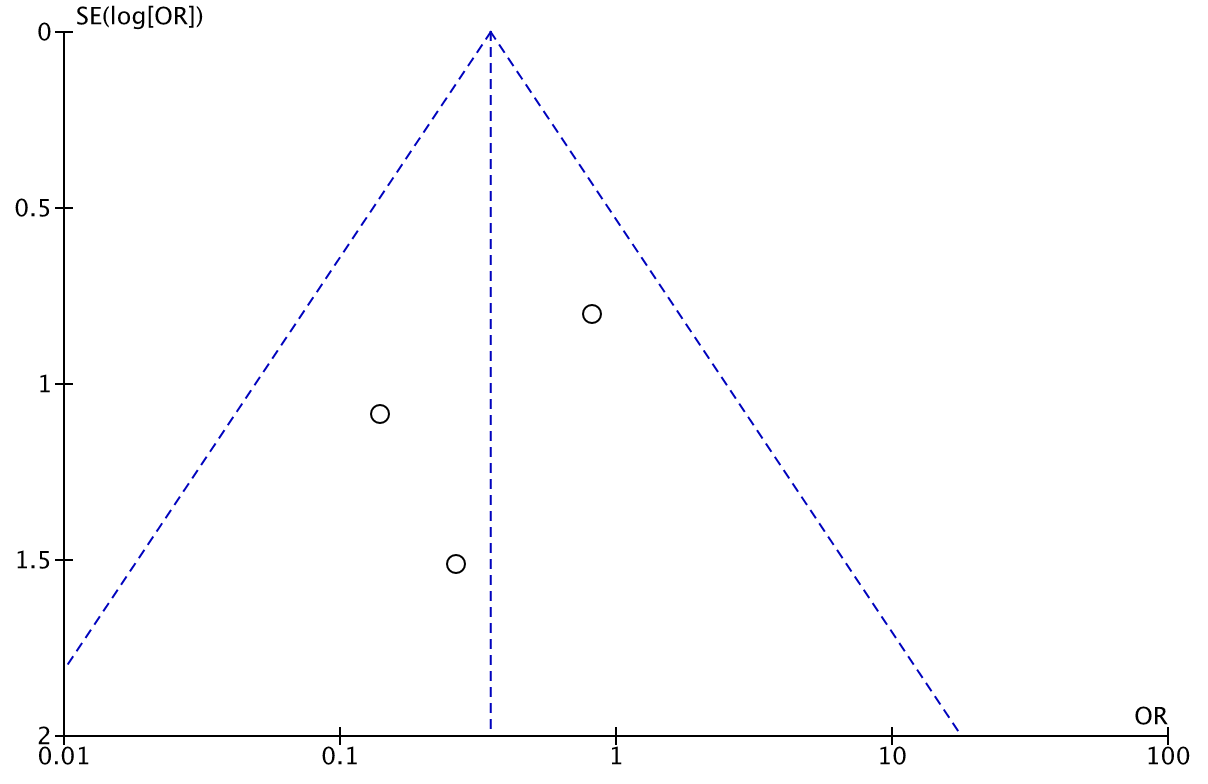


**Figure S14.** Forest and associated funnel plots comparing 5-year overall survival for patients who successfully achieved pathological complete response following neoadjuvant therapies versus those with residual disease.


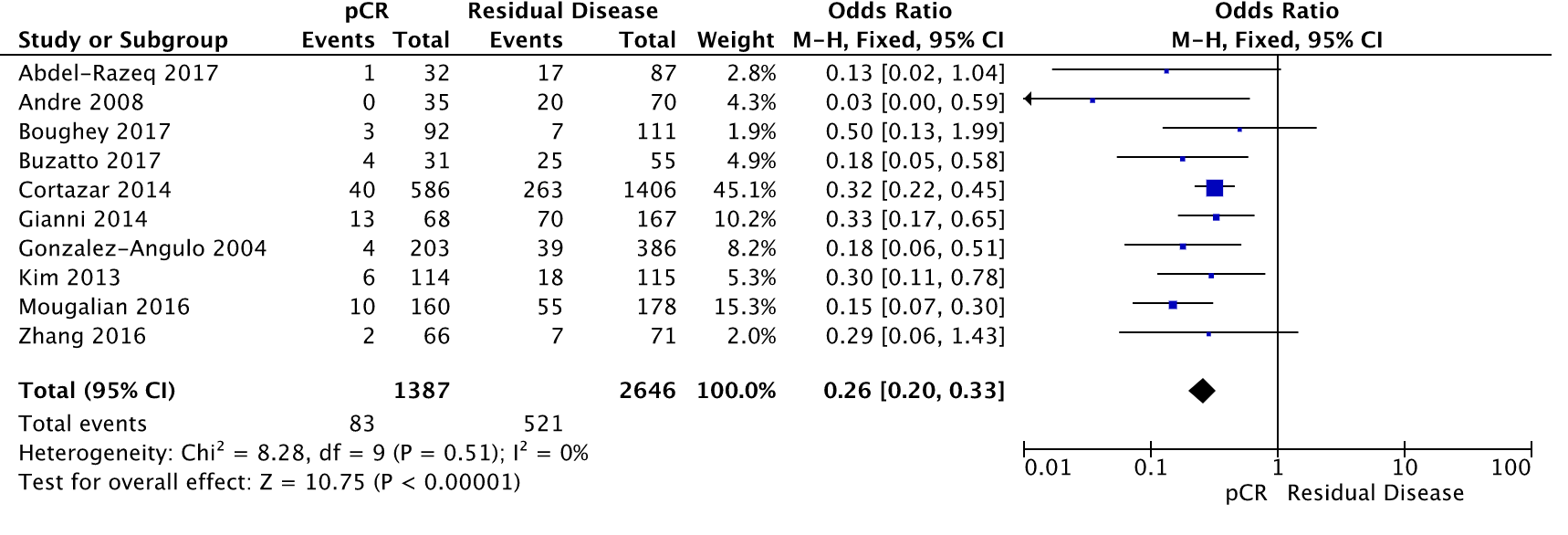

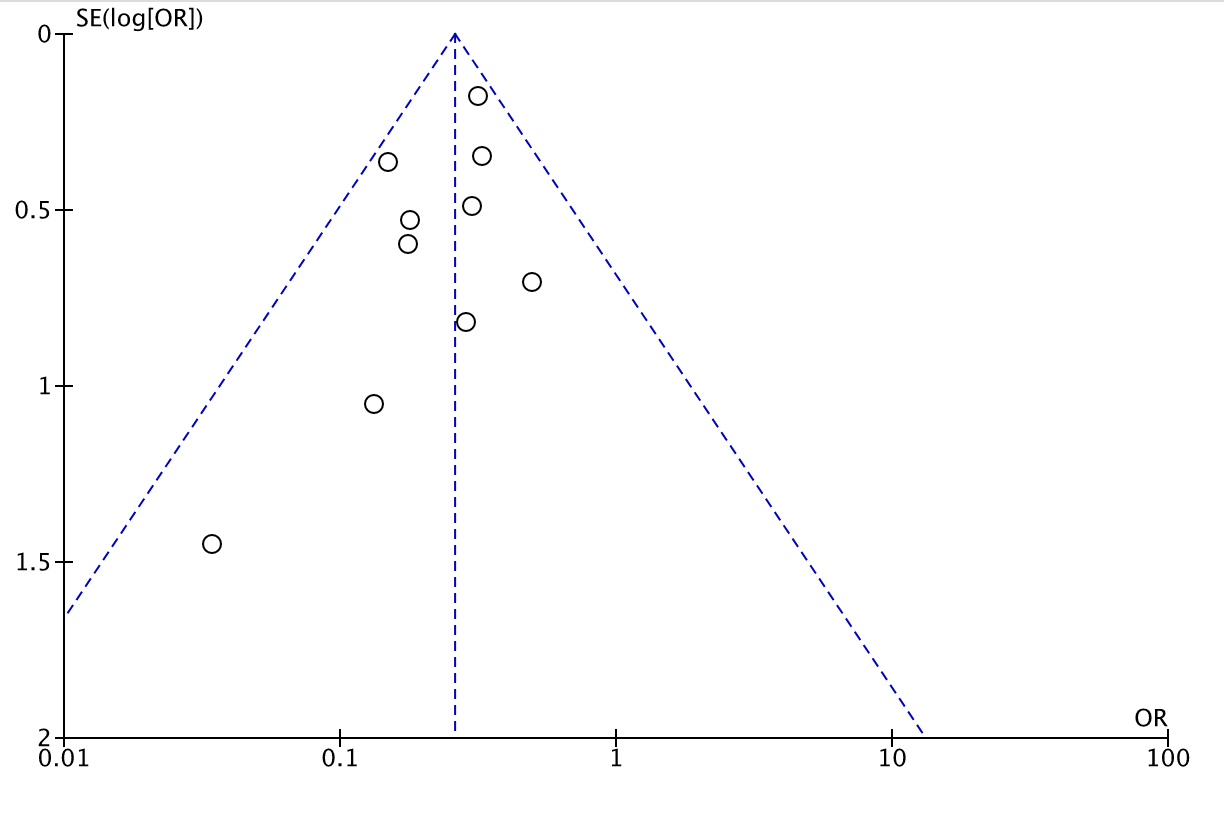

Supplement: zrac028_Supplementary_Data [file zrac028_supplementary_data.zip › Supplementary_material.docx]
